# Functional and gene network analyses of transcriptional signatures characterizing pre-weaned bovine mammary parenchyma or fat pad uncovered novel inter-tissue signaling networks during development

**DOI:** 10.1186/1471-2164-11-331

**Published:** 2010-05-26

**Authors:** Paola Piantoni, Massimo Bionaz, Daniel E Graugnard, Kristy M Daniels, Robin E Everts, Sandra L Rodriguez-Zas, Harris A Lewin, Hurley L Hurley, Michael Akers, Juan J Loor

**Affiliations:** 1 Department of Animal Sciences, University of Illinois, 1207 West Gregory Drive, Urbana, IL, 61801, USA; 2 Department of Animal Science, The Ohio State University, Wooster, OH, 44691, USA; 3 Institute for Genomic Biology, University of Illinois, 1206 West Gregory Drive, Urbana, 61801, USA; 4 Sequenom, Inc., 3595 John Hopkins Court, San Diego, CA 92121, USA; 5 Dairy Science Department, Virginia Tech, Blacksburg, VA, 24061, USA

## Abstract

**Background:**

The neonatal bovine mammary fat pad (**MFP**) surrounding the mammary parenchyma (**PAR**) is thought to exert proliferative effects on the PAR through secretion of local modulators of growth induced by systemic hormones. We used bioinformatics to characterize transcriptomics differences between PAR and MFP from ~65 d old Holstein heifers. Data were mined to uncover potential crosstalk through the analyses of signaling molecules preferentially expressed in one tissue relative to the other.

**Results:**

Over 9,000 differentially expressed genes (**DEG**; False discovery rate ≤ 0.05) were found of which 1,478 had a ≥1.5-fold difference between PAR and MFP. Within the DEG highly-expressed in PAR vs. MFP (n = 736) we noted significant enrichment of functions related to cell cycle, structural organization, signaling, and DNA/RNA metabolism. Only actin cytoskeletal signaling was significant among canonical pathways. DEG more highly-expressed in MFP vs. PAR (n = 742) belong to lipid metabolism, signaling, cell movement, and immune-related functions. Canonical pathways associated with metabolism and signaling, particularly immune- and metabolism-related were significantly-enriched. Network analysis uncovered a central role of *MYC*, *TP53*, and *CTNNB1 *in controlling expression of DEG highly-expressed in PAR vs. MFP. Similar analysis suggested a central role for *PPARG*, *KLF2*, *EGR2*, and *EPAS1 *in regulating expression of more highly-expressed DEG in MFP vs. PAR. Gene network analyses revealed putative inter-tissue crosstalk between cytokines and growth factors preferentially expressed in one tissue (e.g., *ANGPTL1*, *SPP1*, *IL1B *in PAR vs. MFP; *ADIPOQ*, *IL13*, *FGF2*, *LEP *in MFP vs. PAR) with DEG preferentially expressed in the other tissue, particularly transcription factors or pathways (e.g., *MYC*, *TP53*, and actin cytoskeletal signaling in PAR vs. MFP; *PPARG *and LXR/RXR Signaling in MFP vs. PAR).

**Conclusions:**

Functional analyses underscored a reciprocal influence in determining the biological features of MFP and PAR during neonatal development. This was exemplified by the potential effect that the signaling molecules (cytokines, growth factors) released preferentially (i.e., more highly-expressed) by PAR or MFP could have on molecular functions or signaling pathways enriched in the MFP or PAR. These bidirectional interactions might be required to coordinate mammary tissue development under normal circumstances or in response to nutrition.

## Background

As reported by Connor and colleagues [1]: "The mammary gland is a complex organ of various tissue and cell types that will undergo multiple stages of growth, differentiation, secretory activity, and involution during the lifetime of a female mammal". Among the "various tissues" the parenchyma (PAR), which is, in lactating mammary gland, the tissue that synthesizes and secretes milk, and the fat pad (MFP), which is a matrix of connective and adipose tissue surrounding the PAR [2], are considered the most crucial during post-natal development.

Interactions between PAR and MFP during bovine mammary development are still not fully understood. It has been postulated that during mammary development the MFP surrounding PAR exerts proliferative effects on the PAR through secretion of local modulators of growth induced by the impacts of selected systemic hormones (e.g., growth hormone, estrogen) [1,3-5] or growth factors (e.g., IGF-1) [6,7]. It is believed that such an effect occurs because the epithelial tissue that is in direct contact with the MFP has a greater degree of proliferation compared with the more central epithelial tissue [8-10].

Local interaction between PAR and MFP could occur in both directions, i.e., MFP acts on PAR and PAR acts on surrounding MFP [11]. How these tissues could communicate through locally-produced modulators has not yet been studied in the pre-weaning prepubertal bovine mammary gland. Hovey and colleagues [12], using prepubertal ewes, showed that *IGF1 *mRNA expression was greater in MFP cells adjacent to PAR than in MFP cells with no PAR contact, which indicated the existence of a local "diffusible factor" secreted by PAR that could increase the expression of IGF isoform in MFP. Based on those findings, a potential crosstalk between the two tissues was suggested. It was proposed that MFP stimulates PAR and PAR then exerts a positive feedback on the MFP during development [2].

Mammary gland development and tissue interactions have been previously studied using gene expression analysis. For example, in a serial slaughter study [13] it was observed that peak expression of *IGF1 *in MFP and estrogen receptor-α (*ESR1*) in PAR from 100 kg body weight Holstein heifers coincided with peak mammary epithelial cell proliferation [14]. Expression of both genes decreased in mammary tissue in older animals. Regarding tissue interaction, Thorn and colleagues [15] hypothesized that the MFP could impact PAR through inflammation-related proteins, such as TNFα, IL-6, and IL-1β. They showed *in vitro *the inhibitory effect on proliferation of TNFα, but not IL-6 or IL-1β, on epithelial cell proliferation.

Li and colleagues [16] conducted a microarray study exploring the interaction between MFP and PAR in response to estrogen treatment in prepubertal heifers. Results indicated that MFP might affect PAR cell proliferation via the secretion of paracrine stimulators such as the stem cell growth factor precursor C-type lectin domain family 11 member A (*CLEC11A*) and IGF-1. Despite work conducted to date, there is still uncertainty regarding how PAR and MFP tissues interact during mammary development in prepubertal heifers prior to weaning.

In the present study, mammary glands of pre-weaned Holstein heifer calves were harvested at 65 d of age to extract total RNA for microarray analysis. Extensive bioinformatics analysis of microarray data was performed to (1) characterize differences in transcript profiles between mammary PAR and MFP, with the specific aim of uncovering predominant transcriptomic signatures, and (2) uncover predominant signaling molecules (e.g., cytokines and growth factors) in one tissue relative to the other. The latter would allow for the identification of potential targets among genes that are more highly expressed in the other tissue in order to discover novel inter-tissue signaling networks.

## Results

### Coverage of microarray elements in the IPA knowledge base

Over 10,000 oligonucleotides (ca. 76% of total) from the microarray (see details in Additional file 1) were mapped by Ingenuity Pathway Analysis^® ^(**IPA**). Of these, > 7,500 genes were eligible for generating networks and >6,400 genes were associated with a function or pathway. Almost 90% of all annotated genes in our microarray were differentially expressed between PAR and MFP (Table 1 and Additional file 2). Of these, 16.3% had a difference between tissues of > 1.5-fold, with 8.1% being more highly expressed in PAR and 8.2% being more highly expressed in MFP (Table 1 and Additional file 2). Among DEG, 0.6% and 0.8% were 3-fold greater in PAR vs. MFP and MFP vs. PAR, respectively. The data mining analysis was performed on DEG exhibiting ≥1.5-fold difference between tissues. Among DEG, ca. 500 were eligible for generating networks in either PAR or MFP in IPA (Table 1).

**Table 1:** Number of overall differentially expressed genes (DEG, Benjamini-Hochberg FDR ≤ 0.05) and DEG with cut-off of 1.5- and 3.0-fold difference in mRNA abundance between mammary parenchyma and fat pad from pre-weaned Holstein calves (ca. 65 d of age).

	**FD**^**1**^	Number of DEG	Network eligible	Function eligible
Overall tissue effect	< 1.5	9,092		
	1.5	1,478	1,098	986
Expression higher in parenchyma	1.5	736	575	517
	3.0	59		
Expression higher in fat pad	1.5	742	523	469
	3.0	75		

**Reported also is the number of DEG eligible for network and function/pathway analysis in Ingenuity Pathway Analysis^®^**.

^1^Fold-difference between tissues (no difference = 1.0)

### Functional analysis of DEG between PAR and MFP

The IPA analysis results using all DEG with ≥1.5-fold between PAR and MFP are reported in detail in Additional file 3. Briefly, cell movement, cell death, cell growth and proliferation, cell-to-cell signaling and interaction, and tissue development were the top 5 functions among DEG with ≥1.5-fold expression difference. Among canonical pathways, the top 9 were Aryl hydrocarbon receptor signaling, metabolism of xenobiotics by cytochrome P450, propanoate metabolism, pyruvate metabolism, LPS/IL-1 inhibition of RXR function, xenobiotic metabolism signaling, α-adrenergic signaling, p53 signaling, and acute phase response signaling. Interestingly, all of the mentioned pathways were primarily, if not completely, enhanced by genes that were more highly-expressed in MFP vs. PAR. The exception was p53 signaling, which was mostly enriched by genes more highly-expressed in PAR vs. MFP (Additional file 3)

**Figure 1:**
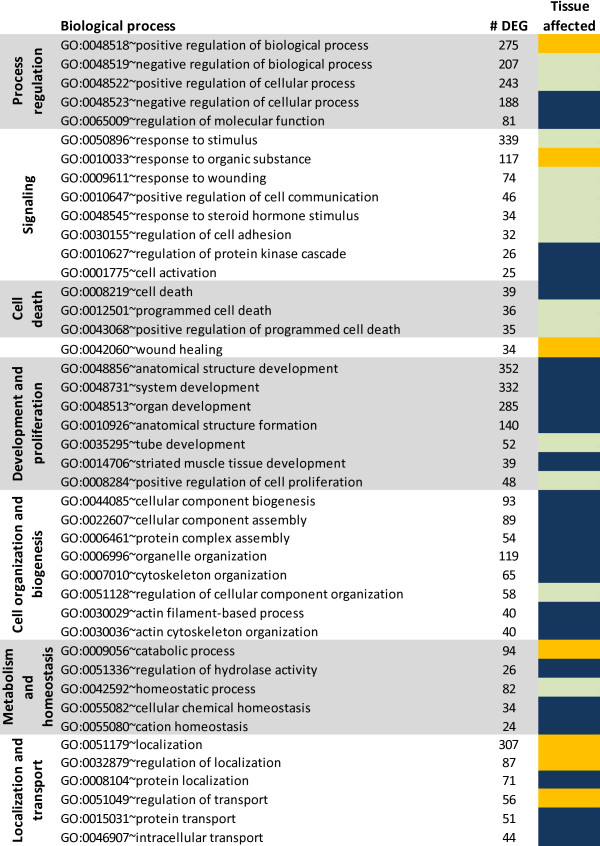
**GO analysis of DEG**. Biological processes significantly-enriched with a Benjamini-Hochberg corrected-*P*-value ≤ 0.05 among all differentially expressed genes (DEG) with ≥ 1.5-fold expression between parenchyma (PAR) and mammary fat pad (MFP). Reported are the Biological processes clustered in pre-selected categories (left column) by the authors to simplify interpretation of the data, the number of DEG for each process (middle column), and main tissue affected by the function (right column): dark blue denotes high enrichment in PAR, orange denotes high enrichment in MFP, and light green denotes same magnitude of enrichment between tissues (see Additional Materials and Methods for explanation). Additional Biological processes and other GO categories (including also Cellular component and Molecular function) are available in Additional file 4.

Results of the most-enriched biological processes from the Gene Ontology (**GO**) analysis that considered all DEG with ≥ 1.5-fold between the two tissues are reported in Figure 1 and in more detail in Additiona file 3. Cell signaling and development were among the most enriched biological processes. Among signaling-associated GO biological process categories (Figure 1) more highly expressed among DEG in PAR relative to MFP, those associated with the protein kinase cascade and cell activation were most predominant. The same types of molecules were enriched in most of development-associated categories. Other functions enriched among DEG with ≥ 1.5-fold in PAR vs. MFP were associated with cell death, cell organization and biogenesis, metabolism and homeostasis, and localization and transport (prevalently protein transport). Biological process categories enriched among DEG ≥ 1.5-fold in MFP vs. PAR were related to wound healing, catabolic processes, and regulation of localization and transport.

### Functions overrepresented in DEG ≥ 1.5-fold in PAR vs. MFP

The main results from the functional analysis with IPA are reported in Table 2. Complete details of the analysis and associated genes are reported in Additional file 3. Among 21 significantly-enriched functions (Benjamini-Hochberg FDR-corrected *P *≤ 0.01), most pertained to functions related to cell development and structure, which became more evident when compared to the functional analysis of DEG more highly expressed in MFP vs. PAR (Table 3). For example, the most-enriched functions among DEG more highly expressed in PAR vs. MFP (with > 130 genes) were cell death, cell growth and proliferation, cellular development, cellular movement, and cell morphology. Enrichment of these opposing processes likely reflects the different cell types within PAR to accommodate the remodeling. Detailed functional analysis of DEG that were more highly-expressed in PAR vs. MFP suggested a greater degree of apoptosis, proliferation/growth/development, movement and adhesion of cells, and morphogenesis/shaping of cells in PAR vs. MFP (Table 2). Overall, angiogenesis, DNA metabolism, and survival of mammals functions were enriched (Table 2) in PAR when compared to MFP. However, detailed analysis of DEG did not indicate induction of gene expression associated with these specific functions.

**Table 2:** Significantly (Benjamini-Hochberg FDR ≤ 0.01) enriched functions among differentially expressed genes (DEG) highly-expressed in parenchyma relative to fat pad using Ingenuity Pathways Analysis^® ^(IPA).

IPA function	*P*-value	# DEG	Main effect on function^1^	Associated functions
Cell Death	3E-13	203	⇑ Apoptosis	
Cellular Growth and Proliferation	2E-11	237	⇑ Proliferation, ⇑ Growth	Tissue Morphology
Cellular Movement	3E-11	137	⇑ Movement of eukaryotic cells	
Cell-To-Cell Signaling and Interaction	2E-09	118	⇑ Adhesion of cells	Tissue Development
Cellular Development	2E-06	146	⇑ Development of eukaryotic cells, lymphatic cells, and blood cells	
Cell Morphology	3E-06	130	⇑ Morphogenesis and shaping of cells	
Cell Cycle	1E-05	107	⇑ Mitosis	
Gene Expression	2E-05	119	⇑ Transcription	
Cardiovascular Sys. Dev. and Funct.	3E-05	57	⇔ Angiogenesis	Organismal Development
Cellular Assembly and Organization	5E-05	116	⇑ Formation of plasma membrane projection	
Hematological Sys. Dev. and Funct.	1E-04	102	⇑ Proliferation of immune cells (leukocytes)	Immune and Lymphatic Sys. Dev. and Funct., Immune response
Nervous Sys. Dev. and Funct.	2E-04	69	⇑ Growth of neurites; ⇑ Migration of Neurons	
Organ Development	2E-04	84	N/A^2^	
Cellular Function and Maintenance	1E-03	21	↑ Cytoskeleton organization; ⇑ Release of Intracellular Store	
Connective Tissue Dev. and Funct.	2E-03	46	⇑ Proliferation and Movement of fibroblasts	
Cell Signaling	2E-03	36	⇑ Quantity of intracellular Ca^2+^	Molecular Transport, Vitamin and mineral metabolism
DNA Replication, Recombination, and Repair	3E-03	76	⇔ DNA synthesis and metabolism; ⇑ Chromatin remodeling	
Hair and Skin Dev. and Funct.	3E-03	25	⇑ Growth of epithelial cells	
Organismal Survival	3E-03	81	⇔ Death and survival of mammalia	
Skeletal/Muscular Sys. Dev. and Funct.	5E-03	14	⇑ Differentiation of bone cells	
RNA Post-Transcriptional Modification	7E-03	5	⇓ Binding of RNA	

**Reported also are the number of genes per function (# DEG), the main effect of those genes within functions (annotation of function from IPA) with the direction of the effect, and other significantly-associated functions (based on effect on function). Details of the analysis are reported in Additional file **2.

^1 ^Major (increase ⇑ or decrease ⇓) or minor (increase ↑ or decrease ↓) effects on function are obtained by the IPA "effect on function" and was considered major if the number of DEG in the specific effect on function denoted as increase/decrease is >10% compared to those DEG that decrease/increase. When no evident direction of the function could be envisaged a ⇔ is reported.

^2 ^No effect on function provided by IPA.

**Table 3:** Significantly (Benjamini-Hochberg FDR ≤ 0.01) enriched functions among differentially expressed genes (DEG) highly-expressed in fat pad relative to parenchyma tissue using Ingenuity Pathways Analysis^® ^(IPA).

IPA function	*P*-value	# DEG	Main effect on function^1^	Associated functions
Lipid Metabolism	5E-11	95	⇑ Synthesis of lipid, ⇑ Oxidation of lipid	Small Molecule Biochemistry
Molecular Transport	5E-08	67	⇑ Transport of lipid (mostly fatty acids)	
Organismal Development	1E-06	79	⇔ Vessel development, ⇓ mass of mammalia	
Cellular Movement	2E-04	114	↑ Migration of eukaryotic cells, ⇑ Invasion of cells	
Tissue Development	2E-04	120	⇔ Adhesion of cells and remodeling of tissue	
Carbohydrate Metabolism	2E-04	63	⇑ Quantity and metabolism of carbohydrates	
Connective Tissue Dev. and Funct.	1E-03	46	⇔ Mass of connective tissue	Tissue Morphology
Cellular Growth and Proliferation	3E-03	164	⇓ Growth and proliferation of cells	
Cell-To-Cell Signaling and Interaction	4E-03	112	⇑ Activation of cell, ⇔ adhesion of cells	
Skeletal/Muscular Sys. Dev. and Funct.	6E-03	34	↑ Proliferation of smooth muscle cells	
Nucleic Acid Metabolism	6E-03	24	⇑ Synthesis of Acyl-CoA and cyclic AMP	
Organismal Functions	7E-02	32	⇑ Locomotion of rodents	

**Reported are the number of genes per function (# DEG), the main effect of those genes in functions (annotation of function from IPA) with the direction of the effect, and other significantly-associated functions (based on effect on function). Details of the analysis are reported in Additional file **2.

^1 ^Major (⇑ or ⇓) or minor (↑ or ↓) effects on function are obtained by the IPA "effect on function" and was considered major if the number of DEG in the specific effect on function denoted as increase/decrease was greater > 10% compare to those DEG that decrease/increase. When no evident direction of the function was envisaged a ⇔ is reported.

**Figure 2:**
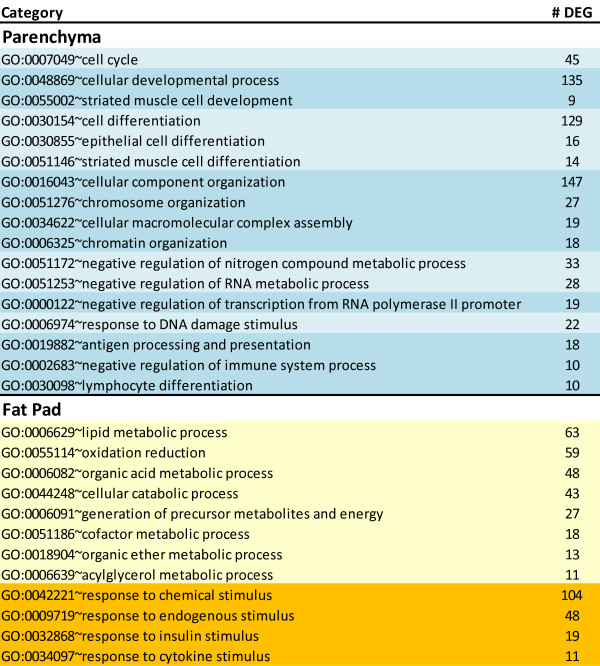
**Comparison of GO enrichment between tissues**. Biological processes significantly-enriched with a Benjamini-Hochberg corrected-*P*-value ≤ 0.05 and uniquely present in differentially expressed genes (DEG) with ≥ 1.5-fold expression in parenchyma (PAR) or mammary fat pad (MFP) relative to the other tissue. Reported are the Biological processes (center column) clustered in pre-selected categories by the authors to simplify interpretation of the data, and the number of DEG for each process (right column). Additional Biological processes and other GO categories (including also Cellular component and Molecular function) are available in Additional file 4 for the same analysis.

The most-enriched biological processes from GO analysis within DEG with ≥ 1.5-fold higher expression in PAR vs. MFP were associated with cell organization (chiefly chromosome/chromatin remodeling), development, differentiation, cell cycle, negative regulation of nucleotide metabolism, negative regulation of transcription, response to DNA damage, and antigen presentation and inhibition of immune system process (Figure 2). Among molecular functions, the most enriched related to binding, particularly protein (i.e., cytoskeletal and enzyme) and nucleotide binding (Additional file 4). Also, functions related to transcription, and particularly repression of transcription, zinc binding, kinases, and cytokine activity were significantly enriched (Additional file 4). The most-enriched cellular components in PAR were of cytosolic origin, particularly components associated with cytoskeleton and intracellular non-membrane-bound organelles. Components related to the nucleus, particularly chromosome allocation were also noted (Additional file 4).

### Functions overrepresented in DEG with ≥ 1.5-fold expression in MFP vs. PAR

The most-abundant functions among DEG that were more highly-expressed in MFP vs. PAR are reported in Table 3. Complete details of the functional analysis and associated genes are reported in the Additional file 3. Metabolic functions related to transport and lipid synthesis and oxidation were among the most significantly-enriched. In addition, IPA analysis of genes more highly expressed in MFP vs. PAR suggested that MFP compared with PAR had a greater degree of cell migration, carbohydrate metabolism, activation of cells, and a lower cellular growth and proliferation (Table 3).

The GO analysis indicated that DEG more highly-expressed in MFP vs. PAR enriched significantly metabolism, particularly lipid biosynthesis, catabolism, and oxidation (Figure 2). A more important role of signaling in MFP compared with PAR was suggested by the significant enrichment of signaling response-related genes, particularly for response to insulin and cytokines (Figure 2). Among cellular functions, the overall analysis indicated a large enrichment of enzyme activity and transport, with the former related to phosphate metabolism and oxidoreductase activity and the latter related to ion (particularly Mg, Fe, and K) and carbohydrate transport with a large enrichment of passive transport-related molecules (Additional file 4). The only significantly-enriched cellular component in MFP was the mitochondria and its related membranes (Additional file 4).

### Main canonical pathways overrepresented among DEG with ≥ 1.5-fold between PAR and MFP

In PAR, the main canonical pathway overrepresented was actin cytoskeleton signaling. This pathway was significantly enriched with an FDR ≤ 0.05 and appeared that it was enhanced among DEG with > 1.5-fold expression in PAR vs. MFP (Table 4). Other pathways appeared to be enriched (Table 4) at a lower level of significance (i.e., FDR ≤ 0.12). Most of those are related to signaling (actin, p53, Wnt/β-catenin, PI3K/AKT) and cell cycle (Table 4). Detailed visualization of the pathways (Additional file 3) suggested an autocrine effect on actin cytoskeleton signaling elicited by FGF (fibroblast growth factor), PDGF (platelet-derived growth factor), and *FN1 *(fibronectin 1), all molecules that were more highly-expressed in PAR vs. MFP (Additional file 2). Among the canonical pathways enriched with a non-corrected *P*-value ≤ 0.01, a detailed analysis of Wnt/β-catenin and PI3K/AKT signaling revealed a potentially crucial role for these in increasing cell cycle activity (e.g., mitosis, Table 2) and apoptosis as well as increasing protein synthesis through mTOR (Additional file 3).

**Table 4:** Top canonical metabolic and signaling (in bold font) pathways uncovered by Ingenuity Pathway Analysis^® ^(Benjamini-Hochberg FDR ≤ 0.05) within differentially expressed genes (DEG) with > 1.5-fold mRNA abundance between parenchyma and fat pad in mammary gland from pre-weaned Holstein heifers.

Pathway	*P*-value	FDR	# DEG
DEG highly-expressed in parenchyma vs. fat pad			
Cell Cycle: G2/M DNA Damage Checkpoint Regulation	0.0025	0.114	7
Nitric Oxide Signaling in the Cardiovascular System	0.0028	0.114	9
**Actin Cytoskeleton Signaling**	**0.0001**	**0.014**	**22**
**p53 Signaling**	**0.0014**	**0.114**	**11**
**Wnt/β-catenin Signaling**	**0.0071**	**0.193**	**15**
**PI3K/AKT Signaling**	**0.0071**	**0.193**	**13**

DEG highly-expressed in fat pad vs. parenchyma^1^			
Propanoate Metabolism	0.0000	0.000	12
Metabolism of Xenobiotics by Cytochrome P450	0.0000	0.001	11
Valine, Leucine and Isoleucine Degradation	0.0000	0.001	11
Butanoate Metabolism	0.0001	0.002	10
Glutathione Metabolism	0.0001	0.002	9
Pyruvate Metabolism	0.0001	0.002	11
Fatty Acid Metabolism	0.0003	0.004	12
Citrate Cycle	0.0003	0.004	7
Glycolysis/Gluconeogenesis	0.0009	0.009	11
Synthesis and Degradation of Ketone Bodies	0.0020	0.016	4
Fatty Acid Elongation in Mitochondria	0.0030	0.022	4
Oxidative Phosphorylation	0.0085	0.055	13
Tryptophan Metabolism	0.0087	0.055	9
Pentose Phosphate Pathway	0.0098	0.056	5
**Mitochondrial Dysfunction**	**0.0000**	**0.000**	**19**
**LPS/IL-1 Mediated Inhibition of RXR Function**	**0.0000**	**0.000**	**20**
**Xenobiotic Metabolism Signaling**	**0.0000**	**0.001**	**23**
**Aryl Hydrocarbon Receptor Signaling**	**0.0001**	**0.001**	**17**
**Acute Phase Response Signaling**	**0.0003**	**0.004**	**19**
**LXR/RXR Activation**	**0.0004**	**0.005**	**10**
**Complement System**	**0.0004**	**0.005**	**7**
**TR/RXR Activation**	**0.0013**	**0.012**	**11**
**α-Adrenergic Signaling**	**0.0013**	**0.012**	**12**
**NRF2-mediated Oxidative Stress Response**	**0.0014**	**0.012**	**18**
**Cardiac β-Adrenergic Signaling**	**0.0014**	**0.012**	**13**
**cAMP-mediated Signaling**	**0.0055**	**0.038**	**13**
**IL-8 Signaling**	**0.0081**	**0.055**	**16**
**IGF-1 Signaling**	**0.0098**	**0.118**	**11**

^1^Reported also are the pathways with Benjamini-Hochberg FDR ≤ 0.12.

The DEG more highly-expressed in MFP vs. PAR had a significant (FDR ≤ 0.05) enrichment of 23 canonical pathways with a large presence of metabolic pathways (11 out of 23 enriched pathways; Table 4). Most of the significantly-enriched canonical pathways were involved in energy utilization, especially utilization of glucose, fatty acids, and several amino acids as sources of energy. Among signaling pathways, most pertained to immune response (e.g., LPS/IL-1 mediated inhibition of RXR function, acute phase response signaling, Complement system) and stress/catabolic response (e.g., mitochondrial dysfunction, NRF2-mediated oxidative stress response, β-adrenergic signaling, cAMP-mediated signaling, aryl hydrocarbon receptor signaling [AHR], xenobiotic metabolism signaling). The overall analysis of canonical pathways suggested an increase in oxidation of organic compounds as also suggested by functional IPA and GO analyses (Table 3 and Figures 1 and 2). The canonical pathway analysis also indicated an inherently-greater predisposition of MFP to mount an immune response compared with PAR.

One noteworthy canonical pathway highlighted by the analysis was IGF-1 signaling, which was enriched at an uncorrected *P*-value ≤ 0.01 (Table 4). The lack of significant enrichment potentially suggests a lower importance of this pathway, but due to the known importance of IGF-1 in mammary development and the likely existence of crosstalk between PAR and MFP [6,13,17,18] we have reported details related to this pathway. Detailed analysis revealed an enrichment of several genes that are downstream (insulin receptor substrate 2 [*IRS2*], v-akt murine thymoma viral oncogene homolog 1 and 2 [*AKT1 *and *2*], protein kinases, Ras) effectors of IGF-1 and its signaling, which overall seems to suggest an increase in cell growth and survival (Additional file 3). However, a number of upstream effectors (e.g., *IGFBP1*, *3*, *5*, *6*) also were enriched in MFP. The above agree with results from Daniels et al. [10] using the same tissues as in the present study. Due to the indication by the functional analysis (Table 3) of an apparent decrease in overall cell proliferation, it could be possible that the IGFBPs exerted some level of control on IGF-1 availability to MFP in these animals

### Transcription factors potentially controlling DEG with ≥ 1.5-fold expression between PAR and MFP

**Figure 3:**
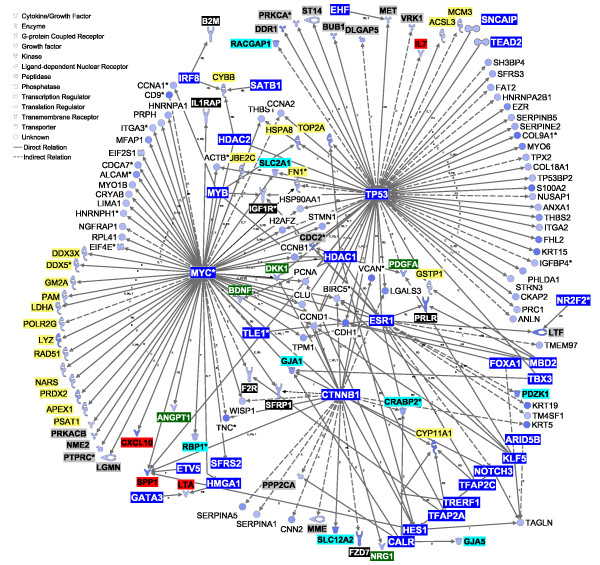
**Regulatory network expression in mammary parenchyma**. Network analysis using differentially expressed genes (DEG) largely expressed (≥ 1.5-fold) in parenchyma (PAR) vs. mammary fat pad (MFP). The interactions shown involve the effect on expression (E), transcription (T), and protein-DNA interactions (PD) between transcription factors (blue background, white font) and other DEG largely expressed (≥ 1.5-fold) in PAR vs. MFP. Molecule types have their background highlighted as follows: enzymes, yellow; cytokines, red; growth factors, dark-green; membrane receptors, black (white font); kinases and phosphatases, gray; transporters, light-blue. The legend for the shape of the objects is reported in the figure. The intensity of the color in the object is proportional to the fold-difference in PAR vs. MFP.

Long-term transcriptomic adaptations of tissues are driven by transcription factors (TF), which sense external stimuli and allow cellular functions to adapt to the specific stimulus. Among DEG more highly-expressed in PAR vs. MFP, IPA identified 76 transcriptional regulators and 2 ligand-dependent nuclear receptors (*ESR1 *and nuclear receptor subfamily 2 group F member 2 [*NR2F2*], Additional file 3). Twenty-nine TF are potentially able to affect the expression of the other 126 DEG more highly expressed in PAR vs. MFP, based on the IPA knowledge base (Figure 3). Most of the genes in the transcriptional networks (Figure 3) are involved in cell cycle and proliferation (e.g., DEG affected by v-myc myelocytomatosis viral oncogene homolog (avian) [*MYC*] and *TP53*). Interestingly, several genes encoding cytokines and growth factors (e.g., *IL7, SPP1, CXCL10*; see description in Table 5) were present in the transcriptional networks, particularly as potential targets of *MYC*, *TP53*, and catenin beta 1 (*CTNNB1*) (Figure 3).

**Table 5:** Parenchyma and fat pad cytokines and growth factors among DEG between tissues (1.5-fold difference in mRNA abundance).

Name	Description	**Biological functions/process**^**1**^	**Type**^**2**^	**FD**^**3**^
DEG highly-expressed in parenchyma vs. fat pad				
***SPP1***	**secreted phosphoprotein 1 (osteopontin)**	**Protein binding**	**Cyt**	**22.1**
***CXCL10***	**chemokine (C-X-C motif) ligand 10**	**Chemokine activity, chemotaxis**	**Cyt**	**3.5**
***PDGFA***	**platelet-derived growth factor alpha polypeptide**	**Actin cytoskeleton organization**	**GrF**	**2.2**
***DKK1***	**dickkopf homolog 1 (Xenopus laevis)**	**Inhibition of Wnt signaling**	**GrF**	**2.1**
***NRG1***	**neuregulin 1**	**Cell-cell communication, murine mammary development**	**GrF**	**2.0**
***BDNF***	**brain-derived neurotrophic factor**	**Anti-apoptosis, axon guidance**	**GrF**	**1.8**
***NTF4***	**neurotrophin 4**	**Regulation of synaptic plasticity**	**GrF**	**1.7**
***ANGPT1***	**angiopoietin 1**	**Angiogenesis, signal transduction**	**GrF**	**1.6**
***IL7***	**interleukin 7**	**Anti-apoptosis, cell-cell signalling**	**Cyt**	**1.6**
***FGF7***	**fibroblast growth factor 7 (keratinocyte growth factor)**	**Positive regulation of epithelial cell proliferation, signal transduction**	**GrF**	**1.6**
***LTA***	**lymphotoxin alpha (TNF superfamily, member 1)**	**TNF receptor binding, immune response**	**Cyt**	**1.6**
***IL1B***	**interleukin 1, beta**	**Immune response, chemotaxis, anti/pro-apoptosis**	**Cyt**	**1.6**
***CCL2***	**chemokine (C-C motif) ligand 2**	**Chemotaxis, immune response, endothelial cell proliferation**	**Cyt**	**1.5**
*CXCL14*	chemokine (C-X-C motif) ligand 14	Chemotaxis, immune response	Cyt	4.4
*CXCL9*	chemokine (C-X-C motif) ligand 9	Chemotaxis, immune response	Cyt	2.3
*VAV3*	vav 3 guanine nucleotide exchange factor	Metal ion binding, protein binding	Cyt	2.2
*HDGF*	hepatoma-derived growth factor	Cell proliferation, regulation of transcription, DNA-dependent	GrF	1.9
*CXCL6*	chemokine (C-X-C motif) ligand 6	Chemotaxis, immune response	Cyt	1.9
*MDK*	midkine (neurite growth-promoting factor 2)	Cell differentiation/proliferation, nervous system development	GrF	1.7

DEG highly-expressed in fat pad vs. parenchyma				
***ADIPOQ***	**adiponectin, C1Q and collagen domain containing**	**Negative regulation of I-kappaB kinase/NF-kappaB cascade, negative regulation of inflammation**	**GrF**	**24.1**
***FGF2***	**fibroblast growth factor 2 (basic)**	**Chemotaxis, positive regulation of angiogenesis and epithelial cell proliferation**	**GrF**	**3.5**
***GRP***	**gastrin-releasing peptide**	**Signal transduction, hormone activity, neuropeptide signaling**	**GrF**	**2.9**
***NOV***	**nephroblastoma overexpressed gene**	**Insulin-like growth factor binding, regulation of cell growth**	**GrF**	**2.5**
***FGF8***	**fibroblast growth factor 8 (androgen-induced)**	**Cell proliferation, signal transduction, induced by androgens in breast cancer cells**	**GrF**	**1.9**
***LEP***	**Leptin**	**Hormone activity, regulation of metabolic process**	**GrF**	**1.9**
***IL13***	**interleukin 13**	**Cell motion, cell-cell signaling, immune response**	**Cyt**	**1.7**
***JAG1***	**jagged 1 (Alagille syndrome)**	**Angiogenesis, cell fate determination, regulation of cell proliferation, activation of Notch signaling pathway**	**GrF**	**1.6**
***IL1A***	**interleukin 1, alpha**	**Immune response, positive regulation of cytokine secretion, pro-angiogenesis**	**Cyt**	**1.6**
*CCL14*	chemokine (C-C motif) ligand 14	Cellular calcium homeostasis, immune response, positive regulation of cell proliferation	Cyt	1.9
*OGN*	Osteoglycin	Protein binding	GrF	1.8
*EDA*	ectodysplasin A	Cell differentiation, immune response, positive regulation of NF-kappaB transcription	Cyt	1.7
*CCL24*	chemokine (C-C motif) ligand 24	Chemotaxis, immune response, cell-cell signaling	Cyt	1.7
*CXCL2*	chemokine (C-X-C motif) ligand 2	Chemotaxis, immune response	Cyt	1.6
*CCL20*	chemokine (C-C motif) ligand 20	Chemotaxis, immune response, cell-cell signalling	Cyt	1.5

Reported in bold font are cytokines and growth factors differentially expressed in one of the two tissues which can potentially interact with highly-expressed DEG in the other tissue as shown in networks reported in Figure 5 and 6.

^1 ^NCBI GO annotation

^2 ^Cytokines (Cyt) and growth factor (GrF)

^2 ^Fold difference (FD) in gene expression between tissues

**Figure 4:**
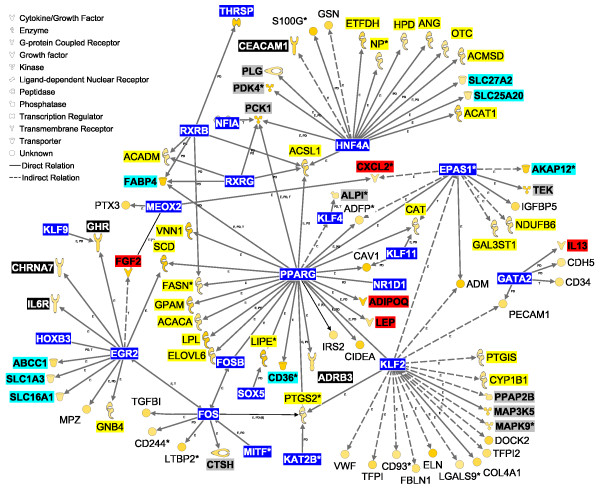
**Regulatory network expression in mammary fat pad**. Network analysis using differentially expressed genes (DEG) largely expressed (≥ 1.5-fold) in mammary fat pad (MFP) vs. parenchyma (PAR). The interactions shown involve the effect on expression (E), transcription (T), and protein-DNA interactions (PD) between transcription factors (blue background, white font) and other DEG largely expressed (≥ 1.5-fold) in MFP vs. PAR. Molecule types have their background highlighted as follows: enzymes, yellow; cytokines, red; growth factors, dark-green; membrane receptor, orange. Highlighted in dark-blue is *THRSP*, which is considered a key lipogenic transcription factor but IPA did not recognize it as such. The legend for the shape of the objects is reported in the figure. The intensity of the color in the object is proportional to the fold-difference in MFP vs. PAR.

Among DEG highly-expressed in MFP vs. PAR, we uncovered 40 TF and 5 ligand-dependent nuclear receptors (peroxisome proliferator-activated receptor γ [*PPARG*], nuclear receptor subfamily 1 group D member 1 [*NR1D1*], progesterone receptor membrane component 2 [*PGRMC2*], and retinoid × receptor beta [*RXRB*] and gamma [*RXRG*]; Additional file 3). *RXRG *(7.6-fold) and *PPARG *(2.8-fold) had the largest differences in expression in MFP vs. PAR (Additional file 3). IPA transcriptional networks indicated that 14 of those TF could potentially affect the expression of 77 more highly expressed genes in MFP vs. PAR (Figure 4). *PPARG*, hepatic nuclear factor 4 alpha [*HNF4A*], Kruppel-like factor 2 (lung) [*KLF2*], early growth response 2 [*EGR2*], and v-fos FBJ murine osteosarcoma viral oncogene homolog [*FOS*] had potentially larger effects on transcription of genes with greater expression in MFP vs. PAR (Figure 4). *PPARG *controls the expression of several enzymes involved in triacylglycerol synthesis and also expression of adipokines, such as adiponectin [*ADIPOQ*] and leptin [*LEP*]. The major functions of the transcriptional networks among more highly expressed DEG in MFP vs. PAR were lipid metabolism (synthesis and transport, mainly) and cellular movement.

### Cytokines and growth factors among DEG with ≥ 1.5-fold expression between PAR and MFP

A total of 9 genes classified as growth factors and 10 genes classified as cytokines had ≥ 1.5-fold greater expression in PAR vs. MFP (Table 5). Among these were osteopontin [*SPP1*] and chemokine (C-X-C motif) ligand 10 [*CXCL10*], which were 22- and 3.5-fold greater in PAR vs. MFP (Table 5). There were 8 genes classified as growth factors and 7 classified as cytokines with greater expression in MFP vs. PAR (Table 5). Among these were *ADIPOQ *and *FGF2*, which were 42- and 3.5-fold greater in MFP vs. PAR.

**Figure 5:**
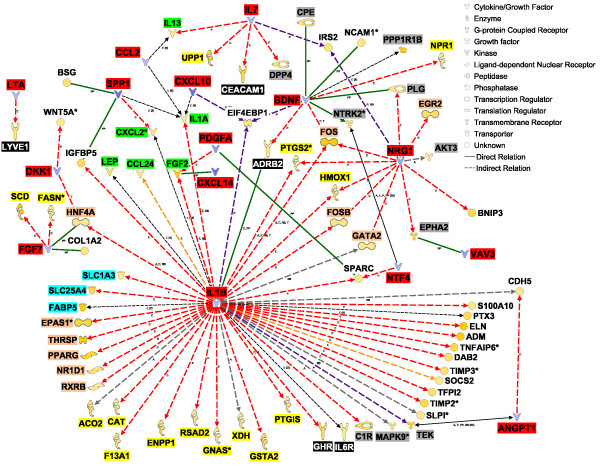
**Cytokine and growth factor enrichment in mammary parenchyma**. Interaction of cytokines and growth factors highly expressed in parenchyma (PAR) relative to mammary fat pad (MFP) with DEG highly expressed in MFP vs. PAR. Objects for cytokines and growth factors largely expressed in PAR vs. MFP are denoted by blue-filled objects. Objects for DEG largely expressed in MFP vs. PAR are depicted in shades of orange. The intensity of the color relates to the fold difference in PAR vs. MFP (blues) of MFP vs. PAR (orange). Molecule types have their background highlighted as follows: enzymes, yellow; cytokines and growth factors potentially secreted by PAR, red; cytokines and growth factors highly-expressed in MFP vs. PAR, green; membrane and G-coupled receptors, black (white font); phosphatase and kinases, gray; transcription factors and nuclear-dependent transcription regulators, dark-pink. All other molecules have white background. The intensity of the shaded color in the object relates to the fold-difference in MFP vs. PAR (orange) or PAR vs. MFP (blue). Red arrows denote effects on gene expression, purple arrows denote activation, dark-violet arrows denote phosphorylation, and green arrows denote protein-protein interactions. Arrow edges are expression (E), activation (A), modification (m), protein-protein interaction (PP), protein-RNA interaction (PR), phosphorylation (P), and translocation (TR). Legend for the shape of the objects is reported in the figure.

**Figure 6:**
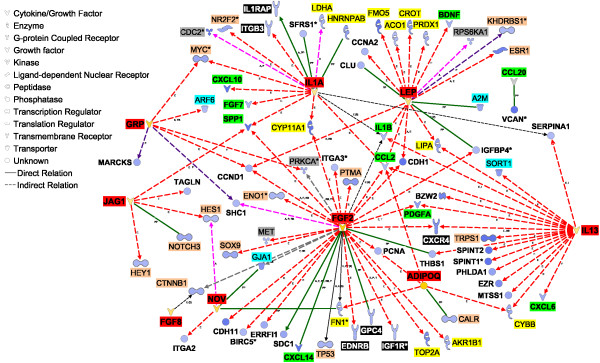
**Cytokine and growth factor enrichment in mammary fat pad**. Interaction of cytokines and growth factors highly expressed in mammary fat pad (MFP) relative to parenchyma (PAR) with differentially expressed genes (DEG) highly expressed in PAR vs. MFP. Objects for cytokines and growth factors largely expressed in MFP vs. PAR have an orange shade. DEG largely expressed in PAR vs. MFP are depicted in blue shades. The intensity of the shaded color in the object relates to the fold-difference in MFP vs. PAR (orange) or PAR vs. MFP (blue). Molecule types have their background highlighted as follows: enzymes, yellow; cytokines and growth factors potentially secreted by MFP, red; cytokines and growth factors highly expressed in PAR vs. MFP light-green; membrane and G-coupled receptors, black (white font); phosphatase and kinases, gray; transcription factors and nuclear-dependent transcription regulators, dark-pink. All other molecules have white background. Red arrows denote effects on gene expression, purple arrows denote activation, dark-violet arrows denote phosphorylation, and green arrows denote protein-protein interaction. Arrow edges are expression (E), activation (A), modification (m), protein-protein interaction (PP), protein-RNA interaction (PR), phosphorylation (P), and translocation (TR). Legend for the shape of the objects is reported in the figure.

Gene network analysis revealed that most of the signaling molecules identified can potentially elicit effects on 1.5-fold DEG between PAR and MFP (Table 5; Figure 5 and 6). Among these is interleukin 1 beta [*IL1B*], which had greater expression in PAR vs. MFP in qPCR analysis (Table S4 in Additional file 1), and had the largest number of connections with DEG that were highly-expressed in MFP vs. PAR (Figure 5). Among the various actions of IL1B, it can affect the expression of several TF, some of which were highly expressed in MFP vs. PAR: *PPARG *and *THRSP *expression, crucial for lipid synthesis, is inhibited by IL1B, whereas endothelial PAS domain protein 1 [*EPAS1*] and *NR1D1 *expression is increased by the same cytokine (Figure 5). IL1B also appears to control the expression of several cytokines and growth factors potentially more actively secreted by MFP compared with PAR (i.e., ≥ 1.5-fold more expressed in MFP vs. PAR), including inhibition of *LEP *and *FGF2*, and activation of *IL1A *(Figure 5).

Other signaling molecules that are likely secreted in greater amounts by PAR than MFP include *FGF7 *and neuregulin 1 [*NRG1*]. Based on IPA annotations, FGF7 decreases the expression of stearoyl-CoA desaturase [*SCD*] while it increases the expression of fatty acid synthase [*FASN*]; whereas, NRG1 appears to regulate the expression of several TF such as *FOS *and *EGR2 *(Figure 5). These two molecules could potentially control the expression of several genes that were more highly-expressed in MFP vs. PAR (Figure 4). In addition, *IL7 *and chemokine (C-C motif) ligand 2 [*CCL2*], potentially released by PAR, may have determined the greater expression of *IL13 *in MFP vs. PAR (Additional file 2).

Among cytokines and growth factors potentially released to a greater extent from MFP compared with PAR, *IL13*, *FGF2*, *IL1A*, and *LEP *had the largest number of potential interactions with DEG that had greater expression in PAR vs. MFP (Figure 6). *FGF2 *may affect a number of biological events through increasing the expression of *IL1B *and *PDGFA*, decreasing the expression of *CCL2*, and decreasing the activation of TP53 in PAR. The transcription factor TP53 might control the expression of many genes in PAR (Figure 3). IL13 also might control the expression of several of the same cytokines and growth factors controlled by FGF2 (Figure 6). LEP might increase expression of several enzymes involved in metabolism (e.g., aconitase 1 [*ACO1*], carnitine O-octanoyltransferase [*CROT*]) as well as decrease the expression of *ESR1*, which seems to have a central role in regulating expression of several other transcription factors (e.g., *CTNN1B*, *FOXA1*, *MYC*) as well as the prolactin receptor (*PRLR*) in PAR (Figure 3). Lastly, it is noteworthy to highlight the cytokine IL1A for its effects on increasing expression of *MYC*, one of the most studied cytokines with a demonstrated role in many functions, chiefly growth and development [19](Figure 3).

### Integrative model of potential interactions between MFP and PAR

**Figure 7:**
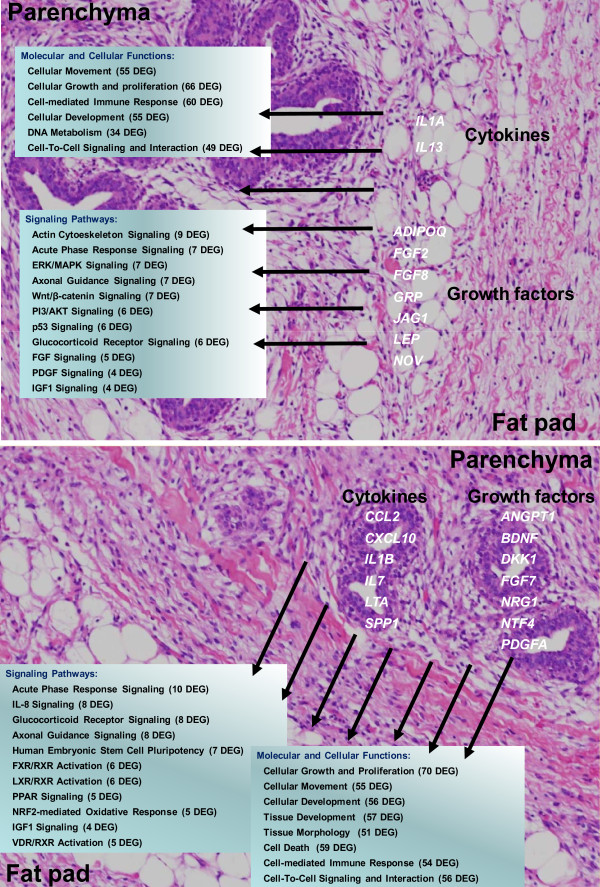
**Model of inter-tissue crosstalk**. Summary of potential effects of cytokines and growth factors predominantly secreted by parenchyma (PAR) or mammary fat pad (MFP) tissue on molecular and cellular functions and signaling pathways suggested by differentially expressed genes (DEG) of the MFP or PAR. The model is based on results from the current study and functions and pathways reported are relative to the functional analysis performed by Ingenuity Pathway Analysis^® ^of interactive networks shown in Figure 5 and 6. See discussion for description of the model.

Results from the functional and gene networks analyses of microarray data were used to develop an integrative model of putative interactions between MFP and PAR (Figure 7). The model highlights growth factors and cytokines that seem to be preferentially expressed in one tissue versus the other. Based on our analysis, which relied on data within the IPA knowledge base, it appears that many of these molecules could interact with genes preferentially expressed in the other tissue and affect a wide range of molecular and cellular functions (Figure 7).

### Microarray data verification through qPCR

Twenty-four out of 25 genes selected were verified via qPCR (Tables S3 and S4 in Additional file 1). These genes were chosen based on their level of expression between tissues as well as their associated biological function. In contrast with microarray data, *IL1B *had greater expression in PAR vs. MFP which was probably due to presence of unspecific binding of the labeled cDNA probe to the oligo on the microarray. Genes confirmed to have greater expression in PAR vs. MFP were *LTF*, *TNC*, *SPP1*, *PRLR*, *CSN3*, *ESR1*, *A2M*, *ACTB*, *CDH1*, *MYC*, *CTNNB1*, and *TP53 *(Additional file 1, Table S3 and S4).

## Discussion

### General considerations

The potential crosstalk between MFP and PAR during bovine mammary development has been recognized for some time (e.g., [2,11,12]). In addition to ovarian steroids [1] and other mammogenic hormones [4], it is believed that various proteins secreted by the MFP (e.g., cytokines, growth factors) act as local regulators of mammary gland growth and morphogenesis [2,11]. In this regard, greater expression of *IGF1 *in MFP and *ESR1 *in PAR tissue has been associated with greater rates of cell proliferation in mammary gland of early prepubertal animals (body weight = 100 kg) than older animals (body weight >100 kg) [13]. In support of this, regardless of nutritional management during the pre-weaning period, Ellis and Capuco [20] demonstrated that there was more epithelial cell proliferation at 2 mo than at 5 or 8 mo age. Those data indicated that mammary gland development and growth is substantial during the pre-weaning period.

Despite the large volume of research regarding development of mammary gland, the functional characterization of mammary PAR and MFP including the underlying gene networks and pathways, as well as putative interactions between both tissues during early development remain to be fully elucidated. The use of transcriptomics analysis via microarrays in combination with bioinformatics analysis can lead to an explosion of biological findings and help uncover potential crosstalk between the PAR and MFP which might affect long-term development of the mammary gland. Only a few studies have attempted to use transcriptomics to study PAR and MFP. For example, Li and colleagues [16] have characterized the transcriptome of MFP and PAR tissue in 5 mo old ovariectomized dairy heifers treated with estrogen and they indentified several genes, most novel, thought to be regulated by estrogen in MFP and PAR, underscoring a crucial role for estrogen in mammary development at this post-weaning stage. We are not aware of similar transcriptomics characterizations in pre-weaned heifers.

In the present study, we have attempted to provide a molecular signature of PAR and MFP using microarrays to uncover genes more highly expressed in one tissue vs. the other. We have corroborated those data through bioinformatics analysis in order to uncover potential molecules involved in the putative crosstalk between PAR and MFP in pre-weaned heifers. Our approach is novel but has the "limitation" common to most bioinformatics tools, as well as transcript annotation, in that the results are based on human and rodent data, as opposed to bovine. However, the large evolutionary similarity between mammalian species (including bovine; [21]) provides reasonable confidence that most of the results in the present study will be suitable for future hypothesis-driven molecular biology experiments.

### Mechanisms of tissue development inferred from functional genomics and pathway analysis

The majority of genes that could be classified as preferentially-expressed in MFP or PAR were associated with cellular growth and proliferation, cellular or tissue development, metabolism, and cellular movement (Tables 2 and 3 and Additional file 3). It was evident from IPA analysis, however, that functional findings encompassed cells other than epithelial cells or adipocytes, e.g., immune and blood/endothelial cells, and fibroblasts (Additional file 3). These responses are not entirely unexpected given our tissue collection protocol, i.e., non-homogeneous cell types within PAR and MFP.

Overall, it was noteworthy that DEG more highly expressed in PAR vs. MFP were enriched significantly and in a greater number (e.g. >230 DEG in cellular growth and proliferation) in the above categories. Furthermore, proliferation and cell cycle were enriched in GO analysis of DEG that were more highly-expressed in PAR vs. MFP (Figure 1 and 2). The evidently larger growth and proliferation of PAR relative to MFP uncovered by transcriptomics agrees with findings by Meyer and colleagues [22] who showed that PAR DNA (mg) was affected more by age than weight of the animal; whereas, the MFP was more affected by nutrition than age. In addition, even though it was not discussed by those authors, calculation of data reported by Meyer and colleagues [22] indicated a greater relative increase (>2-fold from 50 to 350 kg of body weight) in PAR than MFP weight and also greater relative increase in mg PAR DNA than MFP DNA.

In support of greater relative growth in PAR vs. MFP, which is by definition an increase in size or number, the top signaling pathways in DEG more highly expressed in PAR vs. MFP (Table 4; even though considered non-significant at an FDR ≤ 0.05) were all related to cell cycle progression (Table 2). Among those, calcium signaling through PI3K has been associated with cell proliferation and stimulation of quiescent cells to re-enter the cell cycle, thus, initiating mitosis [23]. In addition, the Wnt/β-catenin signaling pathway has been related to control of cell proliferation and differentiation [24,25]. Besides cell growth and proliferation, significantly-enriched functions among the genes with greater expression in PAR vs. MFP were related to apoptosis, cell adhesion, cell organization and biogenesis, and development (Figure 1 and 2, Table 2, and Additional file 3). Those data clearly indicated that besides growth in number and dimension of cells, the mammary PAR underwent a larger degree of re-organization, both within each cell and between cells to form a highly-organized tissue compared to MFP, which is consistent with greater differentiation in PAR compared to MFP (Table 2). The larger degree of organization to support growth in PAR vs. MFP also is supported by the significant enrichment and, apparently, induced Actin Cytoskeleton Signaling (Table 2).

The DEG more highly expressed in MFP vs. PAR indicated that MFP had a more predominant "metabolism-associated" DEG profile compared with PAR, i.e., lipid metabolism, molecular transport (includes lipids and fatty acids), and carbohydrate metabolism were among the most-enriched functions (Table 3, Figure 2). Furthermore, LXR/RXR and TR/RXR activation were among enriched signaling pathways (Table 4). To some extent these findings are not unexpected and could partly be explained by the fact that this tissue is mainly composed of adipocytes, a feature reflected by the higher level of expression of *RXRG *(ca. 8-fold vs. PAR), *PPARG *(ca. 3-fold), *NR1D1 *(ca. 2-fold), and *KLF4 *(ca. 2.5-fold) all of which belong to classical pro-adipogenic pathways (Additional file 2) [26,27].

When we considered the effect on function among DEG more highly expressed in MFP vs. PAR, the large enrichment of tissue development (including connective tissue; Table 3) indicated morphological remodeling. Although the same analysis appeared to suggest that overall cellular growth and proliferation was lower in MFP vs. PAR, it should be noted that several pro-adipogenic factors (e.g., *PPARG*, *NR1D1*, and *KLF4*) were substantially enriched in MFP vs. PAR, some of those known to be associated with continued/sustained mitotic clonal expansion (e.g., *NR1D1*, *KLF4*) of committed pre-adipocytes as well as terminal differentiation, maturation, and hypertrophy of adipocytes (e.g., *PPARG*, *ADIPOQ*) [27]. Because this entire process also is regulated by hormones such as insulin, it is likely that nutritional intervention at this early age leading to altered insulin profiles over the long-term could alter the extent of adipogenesis. This point also is supported by the significant enrichment of response to insulin stimulus in the GO analysis of DEG more highly expressed in MFP vs. PAR (Figure 2).

The enrichment of morphological remodeling in DEG more highly expressed in MFP over PAR was probably due to the increase in size of the cells by accumulation of triacylglycerol, as suggested both by the enrichment of functions related to synthesis and transport of lipid as well as synthesis of acyl-CoA and carbohydrate utilization and also the MFP composition data reported by Daniels et al. for the same heifers [10] (Table 3, Figure 1 and 2). In the present experiment, the weight of MFP of the selected heifers at slaughter averaged 193 g and represented ca. 95% of total mammary gland weight [10], thus, indicating that a large proportion of the mammary tissue in these animals was fat pad. Sinha and Tucker [28] reported a considerable enlargement of the MFP during the pre-weaning phase (birth to 2-3 mo age), and more recent work showed similar results [22,29]. More recently, Meyer and colleagues [22] reported that the increase in MFP weight was more related to enhanced hypertrophy than proliferation, as also evidenced by ratio g of tissue/mg DNA from 50 to 350 kg body weight which was very similar for PAR but increased ca. 3-fold in MFP between 50 kg and 350 kg heifers. Our results suggesting that MFP development was partly related to adipocyte hypertrophy and remodeling appear to be supported by previous findings.

More highly-expressed genes in MFP vs. PAR were preferentially associated with cellular movement (e.g., migration and invasion of cells) and cell-to-cell signaling (e.g., activation and adhesion of cells), functions that are closely related to the immune system (Table 3, Additional file 3). In addition, top signaling pathways among DEG more highly expressed in MFP vs. PAR were primarily related to response to stimuli, e.g., acute phase response, complement system, LPS/IL-1 mediated inhibition of RXR function, IL-8 signaling, and oxidative stress response (Table 4). These data suggest that a significant amount of the DEG more highly expressed in MFP vs. PAR are immune-related genes or that the MFP vs. PAR compartment is more enriched with immune cells (e.g., macrophages) as it has been observed previously in adipose tissue from rodents and humans [30]. Our network analysis (see section below), and previous heifer mammary proteomics data [31], provide evidence that these immune-related pathways in MFP vs. PAR might be biologically relevant in the context of mammary gland development.

We observed a more significant enrichment of IGF-1 signaling among DEG more highly expressed in MFP vs. PAR than *vice versa *(Table 4 and Additional file 3). This pathway was previously related to estrogen and its receptor in 5 mo-old heifers, where increased IGF1 expression was observed after estrogen treatment [5]. In heifers and rodents [32], circulating estrogen seems to act through its receptor and induces MFP cells to secrete IGF-1, which will then act in a paracrine fashion on PAR cells. At least in mice, such a mitogenic effect is observed in spite of low plasma levels of IGF-1 [33]. Our findings, however, do not support an induction of IGF-1 via stimulation of estrogen in MFP compared with PAR. Instead, data suggest that PAR was probably more sensitive to estrogen due to the greater mRNA abundance of *ESR1 *which agrees with a previous study [5] where mRNA expression of *ESR1 *was more predominant (ca. 3-fold greater) in PAR than MFP. Furthermore, no difference in *IGF1 *expression was detected by the microarray analysis or qPCR [10] between the two tissues.

Pathway analysis underscored a more prominent role of IGF-1 signaling in MFP than PAR, which suggests greater sensitivity of MFP to IGF-1 signaling. This is supported by the detailed visualization of the pathway (Additional file 3) which indicates that several key factors in the IGF-1 signaling cascade had greater expression in MFP compared with PAR (e.g., *AKT*, *IRS1*, Ras). Because both *IGFBP5 *and *6 *were more abundant in MFP vs. PAR (Additional files 2 and 3), it could be possible that *IGF1 *activity was reduced [34]. A similar result was found for *IGFBP5 *and *6 *via qPCR in the study of Daniels et al. [10]. As mentioned above, PAR is thought to be affected by local stimulators (e.g., IGF-1) derived from MFP but in our study it appeared that IGF-1 was not a major player in this tissue as it was not among the significantly-enriched pathways (Table 4). Our findings leave open the possibility that other paracrine factors secreted by PAR cells, which can be released in response to circulating estrogen [1], or by the MFP can play a more prominent role during this stage of development in bovine mammary gland.

### Transcriptional network analysis reveals a central role for several transcription regulators in PAR and MFP development

#### Mammary parenchyma

Gene network analysis among DEG that were more highly-expressed in PAR vs. MFP (Figure 3) revealed that the transcription regulators *MYC *(oncogene) and *TP53 *(tumor suppressor) could play central roles. *MYC *has been detected in prepubertal [35] and lactating bovine mammary tissue [36]. Expression of *MYC *mRNA is increased by IGF-1 [37], and a primary response of MYC is to enhance epithelial cell proliferation. Recent evidence showed that *MYC *is essential in mediating Wnt-signaling and subsequent cell proliferation and growth and it also appears to control protein expression through mRNA translation [38,39], all of which can be considered important in PAR development.

*TP53 *can induce cell apoptosis or cell cycle arrest in response to different types of stress, i.e., it is considered to be a central control point of cell transformation and tumorigenesis [40,41]. TP53 activation can induce a transient (cell cycle arrest) or a permanent block of cell proliferation (senescence), or can induce the activation of cell death pathways in response to genotoxic stress [42]. A classical feature of TP53 activation in response to physiological stress (e.g., oxidative stress) leading to DNA damage is the activation of signaling cascades leading to DNA repair, recombination, and the control of DNA replication ultimately protecting cells from endogenous DNA damage [42]. Therefore, on one hand our functional analysis (Figures 1 and 2 and Tables 2 and 4) does not support low cell cycle activity in PAR but, rather, a larger cell cycle activity compared with MFP. On the other hand the more highly expressed DEG in PAR vs. MFP were enriched significantly in functions associated with DNA replication, recombination, and repair (Table 2 and Figure 2). In addition to *MYC *and *TP53*, *CTNNB1 *also may have potentially controlled expression of several genes that were more highly expressed in PAR vs. MFP (Figure 3). The biological significance of *CTNNB1 *in the present experiment is not apparent, but this protein is known to have a pivotal role in alveologenesis during pubertal mammary development in mice [43].

#### Mammary fat pad

Transcriptional gene networks generated among DEG with ≥ 1.5-fold expression in MFP vs. PAR (Figure 4) encompassed a large number of metabolism-related genes, revealing a pattern that would be expected in a classical adipocyte network [26], i.e., several stages of adipogenesis were revealed by some of the overexpressed genes. For example, expression (Figure 4) of *NR1D1 *and *KLF4 *was suggestive of pre-adipocytes undergoing mitotic clonal expansion; whereas, expression of *PPARG *and its putative targets *FASN*, acetyl-Coenzyme A carboxylase alpha [*ACACA*], mitochondrial glycerol-3-phosphate acyltransferase [*GPAM*], elongation of very long chain fatty acids-like 6 [*ELOVL6*], *LPL*, CD36 molecule (thrombospondin receptor) [*CD36*], *FABP4*, acyl-CoA synthetase long-chain family member 1 [*ACSL1*], and *SCD *was indicative of lipid filling that is more characteristic of a mature adipocyte [27]. Not surprisingly, network analysis showed that *PPARG *is a central transcription factor among genes more highly expressed in MFP vs. PAR, and its expression at this stage of development could be essential for adipogenesis and lipid filling in MFP compared with PAR [44] but also for the production of adipokines such as *ADIPOQ *and *LEP *both of which could exert control over tissue inflammation (e.g., through regulation of prostaglandin-endoperoxide synthase 2 [*PTGS2*]).

*PPARG *interacts closely with *RXRG*, *RXRB*, and potentially with lipin 1 (*LPIN1*) [45]. RXR transcription factors participate in the regulation of cholesterol and fatty acid metabolism by interacting with other transcription regulators such as PPARG (i.e., via protein-protein interactions not shown in Figure 4) [44]. *LPIN1 *is one of 3 isoforms that is associated with triacylglycerol synthesis in rodent adipose tissue [46] and fatty acid oxidation in rodent liver [45]. All lipin isoforms were more expressed in MFP vs. PAR (Additional file 2), with *LPIN1 *being the only one having >1.5-fold larger mRNA abundance in MFP vs. PAR. Several other genes in addition to those discussed are considered to play central roles in adipose tissue, including *DGAT2 *(>11-fold higher expressed in MFP vs. PAR). Although not present in the network shown in Figure 4, *DGAT2 *is considered to be a *PPARG*-target gene in non-ruminants and is related to triglyceride synthesis [44]. *THRSP *(potential *RXRB *target gene; Figure 4) is a lipogenic transcription factor and it is synergistically regulated by thyroid hormone and insulin [47,48] as well as long-term via the transcription regulator carbohydrate responsive element binding protein (*MLXIPL *or ChREBP).

Other central transcription factors uncovered by IPA analysis among genes more highly-expressed in MFP vs. PAR were *FOS*, *HNF4A*, *EPAS1*, *KLF2*, and *EGR2 *(Figure 4, Additional file 2). FOS has been previously associated with eukaryotic cell proliferation and differentiation [49,50]. However, the overall transcriptomics functional analysis of DEG more expressed in MFP vs. PAR indicated a lower degree of proliferation/differentiation in MFP vs. PAR (Figures 1 and 2 and Table 3). Transcription of *FOS *is induced with different potency by epidermal growth factor, PDGF, transforming growth factor beta [TGFB], tumor necrosis factor, FGF, IL-1, cAMP, estrogen, and other growth factors that have diverse roles in the cell [49,50]. Thus, in developing bovine mammary tissue this transcription regulator might be essential for MFP development and would provide a functional link with PAR-derived cytokines/growth factors such as IL1B (Figure 5, discussed below). A recent study in mouse mammary cells showed that TGFB regulates the expression of *HNF4A*, which in turn could control cell proliferation [51]. Interestingly, MFP expressed greater mRNA of *TGFB1 *compared with PAR (Additional file 2), which might indicate that, at the moment of harvesting, the influence of PAR-derived TGFB was not relevant for MFP. *KLF2*, was among the TF which potentially could have controlled expression of several DEG more expressed in MFP vs. PAR (Figure 4). The KLF2 protein is an embryonic stem cell-related factor which appears to control pluripotency driven by MYC [52].

Also noteworthy was the higher expression of *EPAS1 *in MFP vs. PAR. *EPAS1 *is preferentially expressed in vascular endothelial cells and plays a pivotal role in the formation of mature vascular tissue [53]. As indicated by functional analysis, angiogenesis was highly-enriched among DEG with ≥ 1.5-fold greater expression in MFP vs. PAR (Table 4); however, the functional analysis did not indicate a greater degree of angiogenesis in MFP compared with PAR. The MFP is essential for ductal morphogenesis in developing mammary tissue, but also it is subject to marked angiogenesis upon stimulation by some factors released from PAR [54]. To our knowledge, EGR2 has not been studied in the context of mammary gland development. Our results suggest that this protein influenced the expression of several genes more highly-expressed in MFP vs. PAR (Figure 4). Among those putative targets, EGR2 could have control over the expression of growth hormone receptor [*GHR*] (Figure 4). The greater expression of *GHR *in MFP vs. PAR agrees with qPCR data from Daniels and colleagues [10].

### Bidirectional crosstalk between tissues inferred from network analysis

The potential effects of MFP-derived growth factors on the developing mammary PAR have been previously studied by several groups with different species [11,55,56]. Similarly, an effect of paracrine factors from PAR on MFP has been suggested from studies of normal mammary epithelial cells [12] and breast tumor epithelial cells [57]. These types of interactions could occur in prepubertal mammary tissue, for example, through growth factors and cytokines such as molecules from the EGF and FGF families [11], which if secreted by either tissue could then act in a paracrine fashion. Thus, to uncover additional factors that could play a role in PAR and MFP development, we evaluated the presence of cytokines and growth factors that might be secreted preferentially by one tissue or the other. The criteria for this analysis was implemented using IPA and was based on the principle that the cytokines and growth factors more highly-expressed in one tissue and, very likely, secreted by it, could potentially affect those DEG that are more highly-expressed in the other tissue (i.e., higher sensitivity to the cytokine or growth factor release in greater amounts by the other tissue) via effects at the level of mRNA expression, functional activation, protein modification, protein-protein interaction, protein-RNA interaction, protein phosphorylation, and protein translocation (see legends in Figure 5 and 6). The multitude of relationships, mostly from rodent and human studies, underscores the importance of these molecules on mammary development.

### Model of putative tissue interactions

Gene network and pathway analysis using IPA allowed us to develop a putative model that would allow both mammary compartments to interact and elicit large-scale changes in the transcriptome and, potentially, tissue function via the synthesis of cytokines and growth factors, many of which have not been studied previously in the context of bovine mammary development. Although our study deals with mRNA expression only, it is assumed that higher expression of cytokines and growth factors in one tissue likely results in greater amount of protein synthesized and very likely more protein secreted. The effect of those signaling molecules can be both at the paracrine, autocrine, as well as endocrine levels. The molecules uncovered by our analysis as being preferentially expressed in PAR compared with MFP or *vice versa *could represent paracrine factors which allow for crosstalk between the two tissues and play important roles in proliferation and development of both epithelial and MFP cells (Figure 7). More importantly, these could be considered an important starting point for future detailed molecular studies of the interaction between PAR and MFP.

Signaling molecules likely released in greater amounts by PAR compared with MFP, through more mRNA abundance in MFP vs. PAR and *vice versa*, and potentially acting on MFP appear to affect, in both tissues, similar functions including cell movement, development, growth and proliferation, cell-mediated immune response, and cell-to-cell signaling and interactions. However, those signaling molecules likely released in greater amounts by PAR compared with MFP can potentially affect cell death in MFP cells; whereas, those released in greater amounts by MFP compared with PAR can potentially affect DNA metabolism in PAR (Figure 7). The potential effects of cytokines and growth factors on cellular growth, development, proliferation, and cell signaling in mammary tissue have been reported for both tissues in a recent review of the literature [4].

Four signaling pathways could have been potentially stimulated reciprocally between the two tissues (Figure 7). Among those were signaling pathways related to acute phase response, axonal guidance, glucocorticoid receptor, and IGF-1 response. The biological significance of the first two is not apparent. Also for the glucocorticod receptor signaling a biological significance is not apparent in our study, nonetheless this pathway has been previously associated with mammary development. In fact, glucocorticoid receptor signaling was associated with the normal development of the virgin mouse mammary gland through stimulation of ductal epithelial cell proliferation [58] and with regulating lobuloalveolar development during pregnancy [59].

The importance of IGF-1 signaling in PAR and MFP development has been discussed above and in previous papers/reviews (e.g., [4,60]). Interestingly, our data suggested that MFP likely affected IGF-1 signaling in PAR not through higher synthesis of IGF-1 but rather by modulating the down-stream signaling. More importantly, our data suggested an active role of PAR in affecting IGF-1 signaling in MFP and based on the number of genes affected and the fact that IGF1 signaling was more significantly enriched in DEG with greater expression in MFP vs. PAR, the stimulation of this pathway appears to be as strong or stronger in MFP than PAR (Table 4 and Figure 7).

Several canonical pathways were uniquely affected in one tissue by signaling molecules released in greater amounts from the other tissue (Figure 7). The cytokines and growth factors preferentially released from PAR compared with MFP seem to influence several nuclear receptors related to lipid metabolism (FXR/RXR, LXR/RXR/PPAR, and VDR/RXR activation), but also maintenance of pluripotency and immune-related pathways (NRF2-mediated oxidative stress) in MFP. The signaling molecules preferentially released from MFP vs. PAR seem to influence PAR signaling pathways with distinctive roles during this stage of development, e.g., actin cytoskeletal signaling (Table 5).

Additional pathways not found to be highly-significant when all DEG were analyzed (Table 4) also seemed to be affected by signaling molecules likely released in greater amounts by MFP compared with PAR, including ERK/MAPK signaling, FGF signaling, and PDGF signaling (Figure 7). ERK/MAPK signaling is related to a broad range of intracellular functions [61] and its role in developing mammary gland is not apparent. FGF signaling is related to the induction of potent mitogenic and angiogenic cellular processes and it is involved in embryonic [62] and postnatal mammary development [63] as well as with tumors of diverse origin, including mammary tumors [64]. PDGF signaling promotes mammary cancer progression and can induce apoptosis of human and murine mammary carcinoma cells when inhibited [65], suggesting a fundamental role in normal mammary differentiation and development. In this regard, Orr Urtreger and Lonai [66] revealed a possible interaction during organogenesis between epithelial and mesenchymal tissue in mice, i.e., PDGFA was expressed in mammary epithelial tissue and its receptor in the surrounding mesenchymal tissue. We are not aware of research in the prepubertal mammary gland of heifer calves that studied these signaling pathways.

## Conclusions

We uncovered specific transcriptomic signatures characterizing genes with large difference in expression between MFP and PAR tissues. Not surprisingly, most of the genes that were more highly expressed in MFP vs. PAR were characteristic of adipose tissue, and those more expressed in PAR vs. MFP were characteristic of an epithelial tissue undergoing expansion and remodeling. Overall, our analyses suggested a large degree of interaction between the two tissues and allowed envisaging a reciprocal influence between the two tissues during this stage of development. This was indicated by the potential effect that the signaling molecules preferentially expressed in PAR vs. MFP and, likely released, have on lipid metabolism-related functions/pathways, which from our data was what distinguished the most those genes more highly expressed in MFP compared with PAR. Similarly, the cytokines and growth factors more highly expressed in MFP compared with PAR potentially affected the functions/pathways related to cell cycle, development, and proliferation in PAR, which our data highlighted as the main functions represented among the genes more highly-expressed in PAR vs. MFP.

Recent efforts have largely focused on MFP as a source of paracrine factors but our study clearly showed that PAR cells could play the same role. Based on the current analysis, the number of cytokines and growth factors that potentially are secreted in greater amounts by each tissue and affect molecules in the other underscores the concept of crosstalk already postulated by several investigators [11,16,67]. Ultimately, these bidirectional interactions might be required to coordinate mammary tissue development under normal circumstances or in response to environmental stimuli, such as nutrition.

Overall, the model generated based on the results from the present experiment predicts a large degree of crosstalk between MFP and PAR with a reciprocal regulation. The main factors at play appear to encompass several cytokines and growth factors preferentially released by PAR including SPP1, CXCL10, PDGFA, DFF1, and NRG1 which are probably slowing down the proliferation of MFP and increasing its lipid accumulation. Concomitantly, cytokines and growth factors released preferentially by MFP such as ADIPOQ, FGF2, GRP, and NOV are probably inducing major re-organization and proliferation of the PAR.

## Methods

### Animals and sampling

Samples used in this study were a subset obtained from a larger experiment [10,68,69]. All animal procedures were conducted under protocols approved by the Virginia Tech Institutional Animal Care and Use Committee. Specific details on feeding, management, and sample collection have been reported previously [10,68,69]. For the current experiment, 19 PAR and 21 MFP samples from 21 Holstein heifer calves (65 d-old; 77.5 ± 2.6 kg BW) representing animals on all diets reported in [10,68,69] were used. Additionally, samples were used to conduct a direct transcriptomics comparison between parenchyma and fat pad. For the present experiment, MFP tissue was harvested from the mammary fat adjacent to the body wall, while PAR tissue was harvested from the macroscopic epithelial portion of the gland. Subsamples of PAR and MFP were snap-frozen in liquid- N_2_, shipped overnight to the University of Illinois, then stored in liquid- N_2 _until use.

### Extraction of RNA, cDNA synthesis, microarrays, and real-time PCR

Details of these procedures are reported in Additional file 1, particularly in Tables S1-S4 and Figure S1. Briefly, PAR and MFP tissues were weighed (~0.5 g) and total RNA extracted using ice-cold Trizol (Invitrogen, Carlsbad, CA). The purity of RNA (A260/A280) was above 1.9. RNA quality was assessed using a 2100 Bioanalyzer (Agilent Technologies, Santa Clara, CA). Samples had a median RNA integrity (RIN) value of 7.7 ± 0.7. cDNA synthesis for microarrays was carried out with a total of 10 μg of RNA (~1 μg/μL). Methods for cleanup and aminoallyl-labeling of cDNA were described previously [70]. Briefly, the aminoallyl-labeled cDNA sample was dried using a speed-vac (Eppendorf Vacufuge^® ^Concentrator, Eppendorf, Westbury, NY) for ~1 h and then resuspended in 4.5 μl 0.1 M sodium carbonate buffer (pH = 9.0). Four and a half microliters of the appropriate Cy dye ester (Cy3 or Cy5; Amersham, Piscataway, NJ) was added to couple the aa-cDNA and incubated for at least 1 h at room temperature. Removal of uncoupled dye was done using the Qiagen PCR Purification Kit.

A bovine oligonucleotide microarray developed at the University of Illinois [71] with > 13,000 bovine oligonucleotides (70-mers) was used to identify large-scale changes in gene expression. Details on the development, annotation, hybridization protocol, and scanning of arrays have been reported previously [71]. In order to increase reliability of data, the following filtering criteria were applied: only slides with ≥ 20,000 (out of > 27,000) spots with a median signal intensity ≥ 3 SD above background in both Cy3 and Cy5 channels and a mean intensity ≥ 400 relative fluorescent units in both Cy3 and Cy5 channels were used. The microarray data have been deposited in NCBI's Gene Expression Omnibus [72] and are accessible through GEO Series accession number GSE20363 http://www.ncbi.nlm.nih.gov/geo/query/acc.cgi?acc=GSE20363.

cDNA to be used in qPCR was synthesized starting from 100 ng total RNA mixed with 1 μg dT18 (Operon Biotechnologies, Huntsville, AL), 1 μL 10 mmol/L dNTP mix (Invitrogen, Carlsbad, CA), 1 μL Random Primers (3 μg/μL, Invitrogen, Carlsbad, CA), and 7 μL DNase/RNase free water. A total of 9 μL of Master Mix composed of 4.5 μL 5× First-Strand Buffer (Invitrogen, Carlsbad, CA), 1 μL 0.1 M DTT (Invitrogen, Carlsbad, CA), 0.25 μL (100 U) of SuperScript™ III RT (Invitrogen, Carlsbad, CA), 0.25 μL of RNase Inhibitor (Promega, Madison WI), 3 μL DNase/RNase free water was added. The reaction was performed in an Eppendorf Mastercycler^® ^Gradient (Eppendorf, Westbury, NY) using the following temperature program: 25°C for 5 min, 50°C for 60 min and 70°C for 15 min. cDNA was then diluted 1:3 with DNase/RNase free water. Four μL of diluted cDNA mixed with 5 μL of SYBR green (Applied Biosystems, Foster City, CA), 0.4 μL of each 10 μM primers, and 0.1 mL of DNase/RNase free water. For real-time RT-PCR each sample was run in triplicate to control reproducibility of the essay and a 4 point relative standard curve (4-fold dilution of cDNA originate from a pool RNA of all samples) plus the non-template control were used. The reactions were performed in an ABI Prism 7900 HT SDS instrument (Applied Biosystems, Foster City, CA) using the following conditions: 2 min at 50°C, 10 min at 95°C, 40 cycles of 15 s at 95°C, and 1 min at 60°C. *PPP1R11*, *MTG1*, *RPS15A *were used as internal control genes to normalize qPCR data [73]. Additional details are reported in Additional file 1.

### Data analyses

Data from a total of 82 microarrays (38 PAR and 44 MFP; 41 samples from 19 animals contributing both PAR and MFP and 3 animals contributing only MFP) were normalized for dye and array effects (i.e., Lowess normalization and array centering) and used for statistical analysis. All data were analyzed using the Proc MIXED procedure of SAS (SAS, SAS Inst. Inc., Cary, NC). To determine differences in mRNA expression between PAR and MFP, the statistical analysis had to be conducted with both PAR and MFP data together, i.e., fixed effects in the model were tissue and dye while random effects included calf and microarray. Raw *P *values for the tissue effect were adjusted using Benjamini and Hochberg's FDR [74]. Differences in relative expression between PAR and MFP were considered significant at an FDR-adjusted *P *= 0.05 for tissue. For a more stringent characterization between the two tissues, a ≥ 1.5-fold difference in mRNA expression was set as threshold among the DEG. Data from qPCR were analyzed using the same statistical model described above. Differences were considered significant at *P *≤ 0.05. The complete statistical output of the microarray analysis is available in Additional file 2.

### Data mining

Data mining was performed using IPA (Ingenuity Systems, Inc., http://www.ingenuity.com) after uploading into the system the entire microarray and qPCR data set with associated FDR and fold differences between PAR and MFP. In IPA, thresholds of FDR = 0.05 and a ≥ 1.5-fold difference were applied to filter significantly affected genes for function, pathway, and network analyses effects. The significance of the association between the dataset filtered by these thresholds and the IPA functions was calculated by IPA using a Benjamini-Hochberg's FDR ≤ 0.01 using the mapped genes on our microarray as background. For canonical pathway analysis we used FDR ≤ 0.05 because the 1.5-fold DEG in PAR vs. MFP did not enrich any pathways at an FDR ≤ 0.01. For a full interpretation of the data generated by the functional analysis in IPA we used the "effect on function" feature in IPA, which allowed determining among those significantly-enriched functions, which specific sub-function/s, tissue, or cellular component/s affected the function and in which direction (i.e., increase/decrease the function). Details of the analysis are reported in Additional file 3 and a summary in Tables 2 and 3.

The gene ontology (GO) analysis was performed using DAVID [75] following the criteria suggested [76]. A Benjamini-Hochberg FDR correction of P-value ≤ 0.05 was set as significant for all categories and all GO terms. The GO analysis was performed in both combined and separate lists of DEG with ≥ 1.5-fold difference between the two tissues. Each separate list of genes with ≥ 1.5-fold difference between the two tissues was used for interpretation of all DEG list using Excel software. Details of the methods are reported in Additional File 1 and full results are reported in Additional file 4 and main findings in Figures 1 and 2.

### Network analyses

Transcriptional networks among transcription factors and DEG with ≥ 1.5-fold difference between PAR and MFP and molecular relationships between cytokines and growth factors preferentially expressed in one tissue vs. the other with DEG more highly expressed in the other tissue were uncovered/built using IPA features and knowledge base.

## Abbreviations

IPA: ingenuity pathway analysis; GO: Gene Ontology; MFP: mammary fat pad; PAR: parenchyma; qPCR: quantitative real-time RT-PCR.

## Authors' contributions

PP performed microarray and qPCR analyses and wrote the manuscript. MB participated in data mining using IPA and GO and wrote the manuscript. DG extracted RNA from tissues. KMD coordinated and helped perform the animal experiment and tissue collection, and helped draft the manuscript. SLR performed microarray statistical analysis. REE and HAL contributed new reagents and tools. RMA helped conceive and design the animal study. WLH helped with data interpretation. JJL designed the microarray experiment and wrote the manuscript. All authors read and approved the final manuscript.

## Supplementary Material

Additional file 1 **Methods and Figure S1**. The file contains additional materials and methods as well as additional results. Extraction and purification of RNA, cDNA synthesis, microarrays, qPCR, Primer design, and GO data analysis accompanied by 4 tables: **Table S1 **titled 'Accession number, sequence, and amplicon size of genes used for qPCR', **Table S2 **titled 'Sequencing results of PCR products of genes designed for this study', **Table S3 **'Genes chosen on fold differences or highly enriched function by DEG between tissues to verify microarray data', and **Table S4 **titled 'Genes chosen on fold differences or highly enriched function by DEG between tissues to verify microarray data'. Click here for file

Additional file 2 **Statistical analysis of genes on the microarray**. The file contains statistical P-values for each gene on the microarray including Benjamini-Hochberg FDR P-values. In addition, separate sheets are included that contain the DEG and DEG sorted by fold-change both in PAR and MFP: 1,478 DEG with FDR ≤ 0.05 and fold difference (FD) >1.5; 736 DEG higher in parenchyma vs. fat pad with FDR ≤ 0.05 and FD >1.5; 742 DEG higher in fat pad vs. parenchyma with FDR ≤ 0.05 and FD >1.5; 59 DEG higher in parenchyma vs. fat pad with FDR ≤ 0.05 and FD >3; 75 DEG higher in fat pad vs. parenchyma with FDR ≤ 0.05 and FD > 3.0. Click here for file

Additional file 3 **Functions and canonical pathways within DEG**. Functions, canonical pathways, effect on function, and transcription factors within DEG (n = ca. 9,000) in both PAR and MFP using IPA (Ingenuity Systems, Inc.). IPA analysis DEG 1.5-fold, without separation between the two tissues and report images of overall functions and pathways affected by DEG with relative -logP-value and list of genes for each significant affected function/pathway: effect on function DEG 1.5-fold, without separation between the two tissues; pathways in all DEG 1.5-fold, reports images of significant affected pathways IPA analysis in DEG PAR, genes highly expressed in PAR vs. MFP and report images of overall functions and pathways affected by DEG with relative -logP-value and list of genes for each significant affected function/pathway; effect on function PAR, genes highly expressed in PAR vs. MFP; pathways in DEG PAR, reports images of significantly affected pathways; IPA analysis in DEG MFP, genes highly expressed in MFP vs. PAR and images of overall functions and pathways affected by DEG with relative -logP-value and list of genes for each significant affected function/pathway; effect on function MFP, genes highly expressed in MFP vs. PAR; pathways in DEG MFP, reports images of significant affected pathways; transcription factor PAR genes highly expressed in PAR vs. MFP; transcription factor MFP, genes highly expressed in MFP vs. PAR. Click here for file

Additional file 4 **GO analysis**. This file contains results from GO analysis of analysis of DEG (overall genes with significant ≥ 1.5-fold difference in expression between PAR and MFP), DEG higher in PAR vs. MFP, and DEG higher in MFP vs. PAR. DEG are classified according to significant biological process, molecular function, and cellular components. Analysis of GO was conducted using DAVID http://david.abcc.ncifcrf.gov:8080/. Click here for file
