# Supplementary material for: Functional and gene network analyses of transcriptional signatures characterizing pre-weaned bovine mammary parenchyma or fat pad uncovered novel inter-tissue signaling networks during development
Source: BMC Genomics. 2010 May 26;11:331. doi: 10.1186/1471-2164-11-331 (PMC2890563; doi:10.1186/1471-2164-11-331)
Supplement: Additional file 1 — Methods and Figure S1. The file contains additional materials and methods as well as additional results. Extraction and purification of RNA, cDNA synthesis, microarrays, qPCR, Primer design, and GO data analysis accompanied by 4 tables: Table S1 titled 'Accession number, sequence, and amplicon size of genes used for qPCR', Table S2 titled 'Sequencing results of PCR products of genes designed for this study', Table S3 'Genes chosen on fold differences or highly enriched function by DEG between tissues to verify microarray data', and Table S4 titled 'Genes chosen on fold differences or highly enriched function by DEG between tissues to verify microarray data'. [file 1471-2164-11-331-S1.DOC]

**Extraction and purification of total RNA**

Parenchymal and fat pad tissues were weighed (~0.5 g) and total RNA extracted using ice-cold Trizol (Invitrogen). RNA was purified using the RNeasy Mini Kit (Qiagen, Valencia, CA, USA). Genomic DNA was removed from RNA with DNase using RNeasy Mini Kit columns (Qiagen, Valencia, CA, USA). RNA concentration was measured using a NanoDrop ND-1000 spectrophotometer (NanoDrop Technologies). The purity of RNA (A260/A280) was above 1.9. RNA quality was assessed using a 2100 Bioanalyzer (Agilent Technologies). Samples had a median RNA integrity value of 7.7 ± 0.7.

**cDNA synthesis**

It was carried out with a total of 10 μg of RNA (~1 μg/μl). RNA was mixed with 2 μl of random hexamer primers (Invitrogen: 3 mg/mL) and 1 μl oligo dT18 (1 μg/μl), and taken to a final volume of 17.78 μl with RNase-free water. The mixture was mixed and incubated at 70 ^o^C for 10 min. After incubation, the tubes were placed on ice for at least 3 min to avoid coiling of RNA. Each sample had 12.2 μL of a master mix added. The master mix was prepared with: 6.0 μl of 5X First Strand Buffer, 3.0 μl of 0.1 M DTT, 0.6 μl of 50X dNTP-dUTP, 0.12 μl of 50 mM aa-UTP, 2.0 μl of SuperScript III RT (200 U/μl), and 0.5 μl of RNase inhibitor. The preparation was mixed and incubated at 23^o^C for 1 min, and then for 46^o^C for 9 h. After the incubation period, samples were kept at 4^o^C to keep integrity of the DNA synthesized. Ten μl of 1 M NaOH was added to remove residual RNA, and then incubated at 65^o^C for 15 min. To neutralize pH and avoid damage of the columns, 10 μl of 1M HCl were added. To remove unincorporated aa-dUTP and free amines, a purification protocol modified from Qiagen Qiaquick PCR purification kit was used. Methods for cleanup and aminoallyl-labeling of cDNA were described by Loor et al. (2005). Briefly, the aminoallyl-labeled cDNA sample was dried using a speed-vac (Eppendorf Vacufuge® Concentrator) for ~1 h and then resuspended in 4.5 μl 0.1 M sodium carbonate buffer (pH = 9.0). Four and a half microliters of the appropriate Cy dye ester (Cy3 or Cy5; Amersham) was added to couple the aa-cDNA and incubated for at least 1 h at room temperature. Removal of uncoupled dye was done using the Qiagen PCR Purification Kit.

**Microarrays**

A bovine oligonucleotide microarray developed at the University of Illinois (Loor et al., 2007) with >13,000 bovine oligonucleotides (70-mers) was used to identify large-scale changes in gene expression. Details on the development, annotation, hybridization protocol, and scanning of arrays have been reported previously (Loor et al., 2007). Methods for microarray hybridizations and scanning were as reported by Loor et al. (2007). Briefly, slides were hydrated, dried, and placed in a UV cross-linker (UV Stratalinker 1800, Stratagene, Agilent Technologies) for ~5 min. Slides were washed with 0.2% SDS solution, rinsed with distilled deionized H_2_O, and placed in warm prehybridization solution for 45 min at 42 ^o^C. Pre-hybridized slides were then rinsed with distilled deionized H_2_O and spin-dried prior to hybridization with labeled cDNA. The same amount of Cy3- or Cy5-labelled cDNA from mammary and a reference standard RNA pool (made of different bovine tissues) were co-hybridized using a dye-swap design (i.e., two microarrays per tissue sample). Slides were incubated for 40 h at 46 ^o^C prior to scanning. In order to increase reliability of data, the following filtering criteria were applied: only slides with ≥20,000 (out of >27,000) spots with a median signal intensity ≥3 SD above background in both Cy3 and Cy5 channels and a mean intensity ≥400 relative fluorescent units in both Cy3 and Cy5 channels were used.

**qPCR**

A portion of the assessed RNA was diluted to 100 mg/L using DNase/RNase free water prior to cDNA synthesis. Sufficient cDNA was prepared at the outset to run all selected genes. Each cDNA was synthesized by RT using 100 ng RNA, 1 μg dT18 (Operon Biotechnologies, AL), 1 μL 10 mmol/L dNTP mix (Invitrogen), 1 μL Random Primers (Invitrogen), and 7 μL DNase/RNase free water. The mixture was incubated at 65 °C for 5 min and kept on ice for 3 min. A total of 9 μL of Master Mix composed of 4.5 μL 5X First-Strand Buffer, 1 μL 0.1 M DTT, 0.25 μL (100 U) of SuperScript^TM^ III RT (Invitrogen), 0.25 μL of RNase Inhibitor (Promega), 3 μL DNase/RNase free water was added. The reaction was performed in an Eppendorf Mastercycler® Gradient using the following temperature program: 25 °C for 5 min, 50 °C for 60 min and 70 °C for 15 min. cDNA was then diluted 1:3 with DNase/RNase free water.

For qPCR analysis, 4 μL of diluted cDNA were combined with 6 μL of a mixture composed of 5 μL 1x SYBR Green master mix (Applied Biosystems), 0.4 μL each of 10 μM forward and reverse primers (Integrated DNA Technologies, Coralville, IA, USA), and 0.2 μL DNase/RNase free water in a MicroAmp™ Optical 384-Well Reaction Plate (Applied Biosystems). Each sample was run in triplicate to control reproducibility of the essay and a 4 point relative standard curve (4-fold dilution) plus the non-template control were used (User Bulletin #2, Applied Biosystems, CA). The reactions were performed in an ABI Prism 7900 HT SDS instrument (Applied Biosystems) using the following conditions: 2 min at 50 °C, 10 min at 95 °C, 40 cycles of 15 s at 95 °C, and 1 min at 60 °C. The presence of a single PCR product was verified by the dissociation protocol using incremental temperatures to 95 °C for 15 s plus 65 °C for 15 s following the last cycle. Complete details regarding qPCR protocol can be found at <http://docs.appliedbiosystems.com/pebiodocs/04364014.pdf>. Data were analyzed with the 7900 HT Sequence Detection Systems Software (version 2.2.3, Applied Biosystems). *PPP1R11*, *MTG1*, *RPS15A* were used as internal control genes to normalize qPCR data. These genes were proven to be stable in mammary parenchyma and fat pad in pre-weaned calves in previous work (Piantoni et al., 2008) and in this same set of samples. For practicality reasons and considering that the HPLF dietary treatment was the one that rendered the most changes, samples from this treatment and Control were used for qPCR verification. Results of qPCR analysis are shown in Suppl. Table 3.

**Primer design**

Primers were designed with Primer Express software version 3.0 (Applied Biosystems, Foster City, CA, USA) using default settings, except for amplicon length (100-150 bp). Primers were designed to span exon junctions when possible and were aligned against public databases using BLAST software available at National Center for Biotechnology Information (NCBI) (Suppl. Table 1). Bovine sequences were used for all selected genes. Evaluation of primers was performed using 3 separate tests: 1) PCR products were visualized in a 2% agarose gel (Invitrogen) stained with ethidium bromide to check for the absence of primer-dimers and the presence of bands at the respective amplicon size (data not shown); 2) PCR products were sequenced at the Core DNA Sequencing Facility of the Roy J. Carver Biotechnology Center at the University of Illinois, Urbana-Champaign, and resulting data were aligned against the NCBI nucleotide database (Suppl. Table 2); 3) lastly, the presence of a single PCR product was verified by the occurrence of a single peak in the dissociation step after qPCR. Primers were used only when they passed all those tests.

**Gene Ontology data analysis**

The GO analysis was performed using David software (freely available at http://david.abcc.ncifcrf.gov/). Out of 13,257 total oligos, DAVID was able to annotate 9,855 genes which were used as background. For the GO analysis bovine, human, mouse, and rat were used as species because, besides bovine, the three non-ruminants species provided the best GO annotation among mammalian species. The individuation of significantly-enriched GO categories was obtained by correction for multiple comparisons using the Benjamini-Hochberg step-down procedure for calculating the FDR in the case of independent tests.

DAVID provided a Benjamini-corrected P-value and the number of genes within each sub-category with the aim of highlighting the biological processes, molecular functions, or cellular components that were significantly enriched by the genes in the list. We up-loaded into DAVID a total of 3 lists: the overall DEG with 1.5-fold difference between parenchyma and fat pad, the DEG with ≥1.5-fold greater expression in parenchyma vs. fat pad, and the DEG with ≥1.5-fold greater expression in fat pad vs. parenchyma. Using the Pivot Table feature in Microsoft Excel software we clustered all the GO categories significantly-enriched in the 3 lists simultaneously. When the significantly-enriched category in the overall gene list was enriched only in parenchyma or fat pad, we concluded that the category was affected in parenchyma or fat pad. When the enriched category in the overall gene list was present in both tissues, we concluded that the category was affecting preferentially one tissue over the other if there was at least a 20% greater number of DEG in one tissue relative to the other.

**Quality control of the samples using mRNA abundance of adipose-specific genes**

Using both microarray and qPCR we wanted to assess if PAR tissue was “contaminated” with MFP, which was essentially an evaluation of tissue dissection quality. Due to the clear adipose-like signature of the MFP we selected to evaluate the expression of adipose-specific genes (*FABP4*, *ADIPOQ*, and *DGAT2*) in both tissues and in all samples (reported in Figure S1 are only samples from control and one of the treatments selected because this diet resulted in the greatest number of DEG, unpublished results). This evaluation was important to ascertain that PAR was relatively free of MFP as the amount of MFP in the mammary compartment grew in weight across experimental diets [10]. Overall, the separation between the tissues was very good, but one sample from MFP (fed HPLF diet, see Materials and Methods) and 1 from PAR (fed HPLF) was removed only from qPCR analysis because it was considered to represent a mixture of tissues when comparing mRNA expression of the tested genes across all samples. The expression of additional genes specific to adipose and mammary (e.g., *SCD*, *CSN2*) also was evaluated using GeneSpring to confirm the above findings with lipogenic genes (data not shown). The remaining samples were deemed appropriate representations of MFP and PAR tissue.

**Table S1.** Accession number, sequence, and amplicon size of genes used for qPCR. Exon/exon junctions are underlined.

| Accession # | Gene Symbol | Primer | Primers (5' – 3') | Amplicon Size (bp) |
| --- | --- | --- | --- | --- |
| CR452243 | *A2M* | F.227 | CACCCAGGACACAGTGGTAGC | 103 |
|  |  | R.329 | CCCTGAAGACTGGATGGTCAC |  |
| BT030480 | *ACTB* | F.258 | ACCAACTGGGACGACATGGA | 149 |
|  |  | R.406 | GTCTCGAACATGATCTGGGTCAT |  |
| BC140488 | *ADIPOQ* | F.214 | GATCCAGGTCTTGTTGGTCCTAA | 131 |
|  |  | R.344 | GAGCGGTATACATAGGCACTTTCTC |  |
| BC123826 | *ADM* | F.335 | CAGAGTTCCGAAAGAAATGGAATAA | 75 |
|  |  | R.409 | GGTAGCTACTGGACTCGCGAAGT |  |
| AY508164.1 | *CDH1* | F.1848 | TCCTGATCTCCCTCCGAACA | 100 |
|  |  | R.1947 | AGACTCACGTTCTTGGTCATTGTACT |  |
| BC102120 | *CSN3* | F.445 | GGCGAGCCTACAAGTACACCTA | 106 |
|  |  | R.550 | GGACTGTGTTGATCTCAGGTGG |  |
| BC119949 | *CTNNB1* | F.1790 | TGCCATTCCACGACTAGTTCAG | 102 |
|  |  | R.1891 | CGGACTCCCTCCACAAACTG |  |
| BT030532 | *DGAT2* | F.389 | CATGTACACATTCTGCACCGATT | 100 |
|  |  | R.488 | TGACCTCCTGCCACCTTTCT |  |
| NM_000125 | *ESR1* | F.1446 | AAGAGGGTGCCAGGCTTTG | 101 |
|  |  | R.1546 | GAGCGCCAGACGAGACCA |  |
| DV778074 | *FABP4* | F.402 | TGGTGCTGGAATGTGTCATGA | 101 |
|  |  | R.502 | TGGAGTTCGATGCAAACGTC |  |
| M13440 | *FGF2* | F.268 | GAGCGACCCACACATCAAACT | 111 |
|  |  | R.378 | AGTAATCTTCCATCTTCTTTCATAGCAA |  |
| CV983373 | *FOS* | F.580 | TCCATGCGTTTTGCTACATCTC | 101 |
|  |  | R.680 | CGTGAAACACACCAGGCTGT |  |
| M37211 | *IL1B* | F.30 | ATTCTCTCCAGCCAACCTTCATT | 100 |
|  |  | R.129 | TTCTCGTCACTGTAGTAAGCCATCA |  |
| BT020625 | *LEP* | F.79 | GGCTTTGGCCCTATCTGTCTTA | 124 |
|  |  | R.202 | GAGACGGACTGCGTGTGTGA |  |
| BC118091 | *LPL* | F.327 | ACACAGCTGAGGACACTTGCC | 101 |
|  |  | R.427 | GCCATGGATCACCACAAAGG |  |
| BC116051 | *LTF* | F.891 | CAAGGCGCAGGAGAAATTTG | 105 |
|  |  | R.995 | AACCCAAGAGCAGAGTCTTTGAA |  |
| BC113343 | *MYC* | F.1613 | GCAGAGCAGCAAAAGCTCAA | 101 |
|  |  | R.1713 | ATTTAGGCGCAAGAGTTCCG |  |
| Y12420 | *PPARG* | F.1356 | GAGCCCAAGTTCGAGTTTGC | 100 |
|  |  | R.1455 | GGCGGTCTCCACTGAGAATAAT |  |
| L02549 | *PRLR* | F.868 | ATAGCATGGTGACCTGCATCC | 91 |
|  |  | R.958 | TCTTCGGACTTGCCCTTCTC |  |
| BC120391 | *RXRG* | F.1270 | GATTACACGTCCACCGAAGCA | 103 |
|  |  | R.1372 | TCCATCTGCATGTCCTTCATTTT |  |
| BC118480 | *S100G* | F.103 | AATATGCAGCCAAAGAAGGTGATC | 100 |
|  |  | R.202 | GTGCTTGGACCCTTCAGCAA |  |
| CK849100 | *SPP1* | F.597 | CTTGGCCTTTGGCGTGAGT | 101 |
|  |  | R.697 | AGGAGATGCATGACGCACC |  |
|  |  |  |  |  |
| AY656814 | *THRSP* | F.631 | CTACCTTCCTCTGAGCACCAGTTC | 151 |
|  |  | R.781 | ACACACTGACCAGGTGACAGACA |  |
| BC126528 | *TNC* | F.4667 | GCTACAGAGGTTCAGTCGGAAAC | 116 |
|  |  | R.4782 | AGAACGACTTCCTTGATTGTACCAT |  |
| BC102440 | *TP53* | F.939 | TGTTTGTGCCTGTCCTGGG | 103 |
|  |  | R.1041 | TGCTCGCTTAGTGCTCCTAGG |  |

**Table S2.** Sequencing results of PCR products from primers of genes designed for this experiment. Best hits using BLASTN (http://www.ncbi.nlm.nih.gov) are shown. Similar information for *ACTB*, *ADIPOQ*, *FABP4*, *LTF*, *SPP1*, *TP53*, *LPL*, and *MYC* was reported previously (Bionaz and Loor, 2007a; Piantoni et al., 2008).

| GenBank ID | Gene | NCBI best hit | Score | % identity | *P*-value | Sequence |
| --- | --- | --- | --- | --- | --- | --- |
| CR452243 | *A2M* | Bos taurus similar to alpha-2-macroglobulin, transcript variant 1 | 102 | 100 | 1.00E-19 | GGACAGCCACTTTCACCAGCGCTAGGAAGGCTGCACAGGTGACCATCCAGTCTTCAGGGGA |
| BC123826 | *ADM* | Bos taurus adrenomedullin (ADM), Mrna, complete cds | 62.6 | 97 | 2.00E-07 | GGATGCCTAAGTGTCGGTGGAAAAAGAGAACTTCGCGAGTCCAGTAGCTAGCCATGATGGGTGTGGTGGAGTGTATGAGAGTA |
| AY508164.1 | *CDH1* | Bos taurus cadherin 1, type 1, E-cadherin (epithelial), mRNA, complete cds | 107 | 98 | 3.00E-21 | CCTTCTAGCAGACTGACACATGGGGCGAGTGTCAATTGGACCATTGAGTACAATGACCAAGAACGTGAAGTCT |
| BC102120 | *CSN3* | Bos taurus kappa casein (CSN3) gene, CSN3-A allele, complete cds | 123 | 100 | 4.00E-26 | GCACTGTAGCTACTCTAGAAGATTCTCCAGAAGTTATTGAGAGCCCACCTGAGATCAACACAGTCCAA |
| BC119949 | *CTNNB1* | Bos taurus catenin (cadherin-associated protein), beta 1, 88kDa | 107 | 98 | 3.00E-21 | CTGTCTGTCCATCAGATACCCAGCGTCGTACATCTATGGGTGGAACACAGCAGCAGTTTGTGGAGGGAGTCCTGA |
| BT030532 | *DGAT2* | Bos taurus diacylglycerol O-acyltransferase homolog 2 (mouse) (DGAT2), mRNA | 87.8 | 96 | 5.00E-15 | GCTGCATGTCGTGTCTCTACTTCCTGGCTGGTGTTTGACTGGACACACCATAGAAAGGTGGCATGGTAGGTCTACTGTTGGGTCGGTGCGTGTGTCTG |
| AY656813 | *ESR1* | Bos taurus estrogen receptor 1 alpha (ESR1) mRNA, partial sequence | 105 | 97 | 1.00E-20 | AAGCATGTGCAGTCAGTCAAGAAATAGCTAGTGGTCTGTGTTTCTTTTAGCCATTGCCTAGCTCCAGA |
| M13440 | *FGF2* | Bos taurus fibroblast growth factor 2 (basic) (FGF2), mRNA | 131 | 98 | 3.00E-28 | AGAGAGAGGGGTTGTGTCTATCAAAGGAGTGTGTGCAAACCGTTACCTTGCTATGAAAGAAGATCGGAAGATTACTACG |
| CV983373 | *FOS* | Bos taurus v-fos FBJ murine osteosarcoma viral oncogene homolog (FOS), mRNA, complete cds | 113 | 100 | 8.00E-23 | TGAGTCCACACGGGGATGCTCGCTTGCAAGTCCTTGAGGCCCACAGCCTGGTGTGTTTCACGGAA |
| M37211 | *IL1B* | Bos taurus interleukin 1 beta (IL1b) mRNA, complete cds | 73.4 | 95 | 8.00E-11 | ACAGCCATGGCACCGTACCTGAACCCATCAACGAAATGATCGGCTTACGTCACAGTGGACAGAGCACAATAGCACCCCC |
| BT020625 | *LEP* | Bos taurus leptin (LEP), mRNA | 131 | 95 | 5.00E-28 | CGCTACTTCCATCTGCAGGTCAGGATGACACCAAAACCCTCATCAAGACAATTGTCACCAGGATCAATGACATCTCACGACACGCAGTCCAGTCTCACG |
| Y12420 | *PPARG* | Bos taurus peroxisome proliferator-activated receptor gamma | 104 | 100 | 4.00E-20 | GACTCGTGATAGATGACAGCGACTTAGCAATATTTATAGCTGTCATTATTCTCAGTGGAGACCGCCA |
| NM_001039726 | *PRLR* | Bos taurus prolactin receptor (PRLR), transcript variant 2, mRNA | 82.4 | 89 | 1.00E-13 | CCAGTTCAGGCCAAATAAAGGATTTGATGTTCATCTGCTGGAGAAGGGCAAGTCCGAAGANTTTTT |
| BC120391 | *RXRG* | Bos taurus similar to Retinoic acid receptor RXR-gamma (Retinoid X receptor gamma) (MGC143016), Mrna | 95.1 | 94 | 4.00E-17 | GCTACGCTGGGTGGCTCATCTTGACAGAGTTCTGACTGAGCTGGTCTCCAAAATGAAGGACATGCAGACTGGTACGACCCCCCGCGCTGTGGGCCTGTTCGGTG |
| BC118480 | *S100G* | Bos taurus calbindin 3, (vitamin D-dependent calcium binding protein) (CALB3), Mrna | 89.7 | 95 | 8.00E-16 | GGAGAGCTGAAGCTACTAGCTTCAGACGGAATTCCCCAGGTTTGCTGAAGGGTCCAAGCACACTGTA |
| AY656814 | *THRSP* | Bos taurus thyroid hormone-responsive protein (THRSP) | 187 | 99 | 1.00E-44 | CGTCATCTGCTCCGTTAGGCTGCCTGCTGCTGTTCACAACTCCTCACTCCTCTTACTAGCTTGGGGTCGGAAGCCAGTGATTCATGAGGGACCACATGTCTGTCACCTGGTCAGTGTGTA |
| BC126528 | *TNC* | Bos taurus similar to Tenascin precursor (TN) (Hexabrachion) (Cytotactin) (Neuronectin) (GMEM) (JI) (Miotendinousantigen) (Glioma-associated-extracellular matrix antigen) (GP 150-225) (Tenascin-C) (TN-C) (MGC140517), mRNA | 122 | 96 | 2.00E-25 | GAGACCTCCCCGGGCATCTGTCACTGGTTACCTATTGGTGTATGAATCCGTGGATGGTACACATCTAAGGAAGTCAGTTCTACTGCT |

**Table S3.** Genes chosen on fold differences or highly enriched function by DEG between tissues to verify microarray data. Here are reported DEG highly expressed in parenchymal vs. fat pad tissue. All genes here reported were verified.

| **Chosen on fold change** | | | | |
| --- | --- | --- | --- | --- |
| **Gene** | **Description** | **Microarray*** | **qPCR**** | **Function (NCBI)** |
| *A2M* | Alpha-2-macroglobulin | 1.98 | 1.56 | Protease inhibitor and cytokine transporter |
| *CSN3* | Casein kappa | 4.85 | 15.82 | Milk protein |
| *ESR1* | Estrogen receptor 1 | 2.82 | 2.61 | Mediates estrogen activity |
| *LTF* | Lactotransferrin | 11.41 | 20.37 | Non-specific antimicrobial activity, regulation of iron homeostasis, anti-inflammatory activity, regulation of cellular growth and differentiation |
| *PRLR* | Prolactin receptor | 6.34 | 3.91 | Mediates prolactin activity |
| *SPP1* | Secreted phosphoprotein 1 (osteopontin, bone sialoprotein I, early T-lymphocyte activation 1) | 22.07 | 10.92 | Cytokine activity and tissue development |
| *TNC* | Tenascin C (hexabrachion) | 7.25 | 4.32 | Tissue morphology and development, cellular movement |
| **Chosen on Function** | | | | |
| **Gene** | **Description** | **Microarray*** | **qPCR**** | **Function (NCBI)** |
| *ACTB* | Actin, beta | 1.98 | 1.45 | Cell motility, structure, and integrity |
| *CDH1* | Cadherin 1, type 1, E-cadherin (epithelial) | 5.02 | 15.28 | Proliferation control |
| *CTNNB1* | Catenin (cadherin-associated protein), beta 1, 88kDa | 1.83 | 1.54 | Adhesion between cells, regulation of normal cell growth and behavior |
| *MYC* | V-myc myelocytomatosis viral oncogene homolog (avian) | 2.02 | 1.58 | Cell progression, apoptosis, and cellular transformation |
| *TP53* | Tumor protein p53 (Li-Fraumeni syndrome) | 1.71 | 2.55 | Tumor suppressor by regulation of genes that induce cell cycle arrest, apoptosis, senescence, DNA repair, or changes in metabolism |

* FDR *P* < 0.05

** *P*-value < 0.01

**Table S4.** Genes chosen on fold differences or highly enriched function by DEG between tissues to verify microarray data. Here are reported DEG highly expressed in fat pad vs. parenchyma tissue. All genes here reported were verified except *IL1B*.

| **Chosen on fold change** | | | | |
| --- | --- | --- | --- | --- |
| **Gene** | **Description** | **Microarray*** | **qPCR**** | **Function (NCBI)** |
| *ADIPOQ* | Adiponectin, C1Q and collagen domain containing | 24.1 | 13.07 | Lipid metabolism, and other metabolic and hormonal processes |
| *DGAT2* | Diacylglycerol O-acyltransferase homolog 2 (mouse) | 11.74 | 14.82 | Synthesis of triglycerides |
| *FABP4* | Fatty acid binding protein 4, adipocyte | 34.61 | 11.08 | Long-fatty acids uptake, transport, and metabolism |
| *LPL* | Lipoprotein lipase | 5.47 | 12.49 | Triglyceride hydrolase and ligand factor for receptor-mediated lipoprotein uptake |
| *S100G* | S100 calcium binding protein G | 11.95 | 16.09 | Calcium transport activity |
| *THRSP* | Thyroid hormone responsive (SPOT14 homolog, rat) | 9.37 | 8.58 | Control of lipid metabolism |
| **Chosen on function** | | | | |
| **Gene** | **Description** | **Microarray*** | **qPCR**** | **Function (NCBI)** |
| *ADM* | Adrenomedullin | 6.77 | 7.48 | Hypotensive functions |
| *FGF2* | Fibroblast growth factor 2 (basic) | 3.50 | 4.69 | Broad mitogenic and angiogenic activities |
| *FOS* | V-fos FBJ murine osteosarcoma viral oncogene homolog | 4.13 | 1.31 | Regulation of cell proliferation, differentiation, and transformation |
| *IL1B* | Interleukin 1, beta | 1.63 | -1.58 | Mediator of inflammatory response, involved in cell proliferation, and apoptosis |
| *LEP* | Leptin (obesity homolog, mouse) | 1.90 | 12.82 | Inhibition of food intake, regulation of energy expenditure to maintain constancy of adipose mass, regulation of immune and inflammatory responses |
| *PPARG* | Peroxisome proliferator-activated receptor gamma | 2.81 | 3.17 | Regulation of adipocytes differentiation |
| *RXRG* | Retinoid X receptor, gamma | 7.65 | 15.42 | Mediates the antiproliferative effects of retinoic acid |

* FDR *P* < 0.05

** *P*-value < 0.01

**Figure S1.**  Raw qPCR data to verify fat pad infiltration of parenchyma between experimental diets (control and high-protein:low-fat milk replacer (280 g/kg crude protein, 200 g/kg crude fat, fed at 951 g DM/d). Samples from PAR of calf 19 and MFP of calf 11 were deemed to contain a mixture of PAR and MFP, thus, were removed from qPCR statistical analysis.

**References**

Loor, J. J., R. E. Everts, M. Bionaz, H. M. Dann, R. Oliveira, D. E. Morin, S. L. Rodriguez-Zas, J. K. Drackley, H. A. Lewin. 2007. Nutrition-induced ketosis alters metabolic and signaling gene networks in liver from periparturient cows. Physiol. Genomics. 32:105–116.

Piantoni, P., M. Bionaz, D. E. Graugnard, K. M. Daniels, R. M. Akers, and J. J. Loor. 2008. Gene expression ratio stability evaluation in prepubertal bovine mammary tissue from calves fed different milk replacers reveals novel internal controls for quantitative polymerase chain reaction. J Nutr 138(6):1158-1164.
